# Downregulation of *GAUT12* in *Populus deltoides* by RNA silencing results in reduced recalcitrance, increased growth and reduced xylan and pectin in a woody biofuel feedstock

**DOI:** 10.1186/s13068-015-0218-y

**Published:** 2015-03-12

**Authors:** Ajaya K Biswal, Zhangying Hao, Sivakumar Pattathil, Xiaohan Yang, Kim Winkeler, Cassandra Collins, Sushree S Mohanty, Elizabeth A Richardson, Ivana Gelineo-Albersheim, Kimberly Hunt, David Ryno, Robert W Sykes, Geoffrey B Turner, Angela Ziebell, Erica Gjersing, Wolfgang Lukowitz, Mark F Davis, Stephen R Decker, Michael G Hahn, Debra Mohnen

**Affiliations:** Department of Biochemistry and Molecular Biology, University of Georgia, B122 Life Sciences Bldg., Athens, GA 30602 USA; Department of Plant Biology, University of Georgia, 2502 Miller Plant Sciences, Athens, GA 30602 USA; Complex Carbohydrate Research Center, University of Georgia, 315 Riverbend Road, Athens, GA 30602 USA; DOE-BioEnergy Science Center (BESC), Oak Ridge, USA; Bioscience Division, Oak Ridge National Laboratory, Oak Ridge, TN 37831 USA; ArborGen Inc., 2011 Broadbank Ct, Ridgeville, SC 29472 USA; National Renewable Energy Laboratory, 15013 Denver West Parkway, Golden, CO 80401-3305 USA

**Keywords:** Biofuel, Growth, Pectin, *Populus*, Saccharification, Secondary cell wall, Xylan, Wood development

## Abstract

**Background:**

The inherent recalcitrance of woody bioenergy feedstocks is a major challenge for their use as a source of second-generation biofuel. Secondary cell walls that constitute the majority of hardwood biomass are rich in cellulose, xylan, and lignin. The interactions among these polymers prevent facile accessibility and deconstruction by enzymes and chemicals. Plant biomass that can with minimal pretreatment be degraded into sugars is required to produce renewable biofuels in a cost-effective manner.

**Results:**

GAUT12/IRX8 is a putative glycosyltransferase proposed to be involved in secondary cell wall glucuronoxylan and/or pectin biosynthesis based on concomitant reductions of both xylan and the pectin homogalacturonan (HG) in *Arabidopsis irx8* mutants. Two GAUT12 homologs exist in *Populus trichocarpa*, *PtGAUT12.1* and *PtGAUT12.2*. Knockdown expression of both genes simultaneously has been shown to reduce xylan content in *Populus* wood. We tested the proposition that RNA interference (RNAi) downregulation of *GAUT12.1* alone would lead to increased sugar release in *Populus* wood, that is, reduced recalcitrance, based on the hypothesis that GAUT12 synthesizes a wall structure required for deposition of xylan and that cell walls with less xylan and/or modified cell wall architecture would have reduced recalcitrance. Using an RNAi approach, we generated 11 *Populus deltoides* transgenic lines with 50 to 67% reduced *PdGAUT12.1* transcript expression compared to wild type (WT) and vector controls. Ten of the eleven RNAi lines yielded 4 to 8% greater glucose release upon enzymatic saccharification than the controls. The *PdGAUT12.1* knockdown (*PdGAUT12.1*-KD) lines also displayed 12 to 52% and 12 to 44% increased plant height and radial stem diameter, respectively, compared to the controls. Knockdown of *PdGAUT12.1* resulted in a 25 to 47% reduction in galacturonic acid and 17 to 30% reduction in xylose without affecting total lignin content, revealing that in *Populus* wood as in *Arabidopsis*, GAUT12 affects both pectin and xylan formation. Analyses of the sugars present in sequential cell wall extracts revealed a reduction of glucuronoxylan and pectic HG and rhamnogalacturonan in extracts from *PdGAUT12.1*-KD lines.

**Conclusions:**

The results show that downregulation of GAUT12.1 leads to a reduction in a population of xylan and pectin during wood formation and to reduced recalcitrance, more easily extractable cell walls, and increased growth in *Populus*.

**Electronic supplementary material:**

The online version of this article (doi:10.1186/s13068-015-0218-y) contains supplementary material, which is available to authorized users.

## Background

*Populus* is a woody feedstock for biofuel and bioproduct formation. The major challenge of using woody feedstock as a source for biofuels is the rigid cell wall, which is recalcitrant to degradation by bacterial and fungal enzymes [1-3]. The identification of genes and proteins involved in the formation of secondary cells wall is necessary to understand and overcome the recalcitrance of woody feedstocks. Towards this aim, we have manipulated the expression of putative ‘recalcitrance’ genes in *Populus* for use in studying the genetic basis of recalcitrance in this biomass feedstock.

Wood formation in *Populus* starts with the differentiation of secondary cell walls. Cellulose, hemicellulose, and lignin are the three major components of *Populus* secondary walls, with pectin being a minor component. In *Populus* wood, the hemicelluloses are largely xylans which provide 18 to 28% of the total dry weight [4]. Xylans are polysaccharides with linear backbones of β-(1 → 4)-linked d-xylosyl residues. The major xylan in dicot wood, glucuronoxylan (GX), is decorated with side chains of *O*-2-linked α-d-glucuronic acid (GlcA) and/or 4-*O-*methyl-α-d-glucuronic acid (MeGlcA). The xylosyl backbone in secondary wall xylan is also highly acetylated at C-2 and C-3.

Multiple types of *Arabidopsis* xylan synthesis mutants [5], including xylan backbone mutants *irx9 irx9-L* [6-9], *irx14 irx14-L* [9-11], *irx10 irx10*-*L* [12,13], and *irx15 irx15*-*L* [14,15], have been extensively studied in an effort to understand xylan biosynthesis. The recovery of xylan xylosyltransferase activity from heterologously expressed Arabidopsis IRX10-L [16] and *Plantago ovate* IRX10 [17], and the demonstration of xylan acetyltransferase activity from heterologously expressed Arabidopsis ESK1/TBL29 [16], confirmed a role for these enzymes in xylan backbone synthesis and acetylation, respectively. *Arabidopsis* xylan substitution mutants *gux1* and *gux2* have reduced α-glucuronidation of the xylan backbone [18,19] while the level of methylation of the GlcA residues is reduced in *gxmt1* mutants [20]. The respective genes have been shown to encode functional xylan glucuronosyltransferases [18] and xylan 4-*O*-methyltransferases [20]. Additional *Arabidopsis* mutants have also been identified that have defects in both xylan and other cell wall polymers. For example, xylan and cellulose deposition are affected in *irx7* (*fra8*) and *irx7-L* (F8H) [10,21,22] while *qua1* [23-25], *parvus-3* [10,26-28], and *irx8/GAUT12* [8,29,30] have defects in both pectin and xylan.

The *Arabidopsis irx8* mutant has been extensively characterized in Arabidopsis [5]. The *IRX8/GAUT12* gene belongs to the *GAUT1* (GAlactUronosylTransferase1)-related gene family. The *GAUTs* constitute one clade of the glycosyltransferase 8 (GT8) family [30-33]. The family name, GAUT, originated with the identification of Arabidopsis galacturonosyltransferase 1 (*GAUT1*). GAUT1 is a pectin biosynthetic homogalacturonan (HG):α-1,4-galacturonosyltransferase (GalAT) that functions in an HG:GalAT protein complex with GAUT7 [34-36]. Arabidopsis *GAUT12*/*irx8* has highest expression in cells with secondary walls, and the encoded protein has 61% amino acid sequence similarity with GAUT1. GAUT12 is predicted to be a type II membrane protein targeted to the Golgi. The *irx8/gaut12* mutation leads to a reduction in GX; however, microsomes from *irx8* mutant stems did not show any reduction in xylan XylT activity [7,10] or xylan GlcAT (glucuronosyltransferase) activity [7] compared to microsomes from wild type (WT). Structural analysis of cell walls from *irx8 Arabidopsis* mutant plants identified a dramatic reduction in GX and in a tetrasaccharide sequence β-d-Xyl*p*-(1-3)-α-l-Rha*p*-(1-2)-α-d-Gal*p*A-(1-4)-d-Xylp located at the reducing end of xylan [8,29]. A concomitant reduction in a subfraction of the pectin HG was also identified in walls of *Arabidopsis irx8* mutants compared to WT leading to the hypotheses that GAUT12 is involved in either xylan or HG synthesis [29].

*Populus* has two orthologs of the *Arabidopsis GAUT12* gene: *PtGAUT12.1* (*POPTR_0001s44250*, Genemodel V2.0, Phytozome 8.0, http://www.phytozome.net; *Potri.001G416800*, Genemodel V3.0, Phytozome 10.0, http://phytozome.jgi.doe.gov) and *PtGAUT12.2* (*POPTR_0011s13600*, Genemodel V2.0, Phytozome 8.0; *Potri.011G132600*, Genemodel V3.0, Phytozome 10.0). Downregulation of both genes simultaneously in transgenic *P. trichocarpa* RNA interference (RNAi) lines (*PtrGT8D1/POPTR_0001s44250/Potri.001G416800* and *PtrGT8D2/POPTR_0011s13600/Potri.011G132600*) led to a major reduction in xylan and an increase in lignin in the stem, along with reduced wall thickness in fiber cells [37]. Based on these results, a role for *PtrGT8D* in GX biosynthesis in *Populus* wood has been suggested [37]. The irregular xylem and dwarf phenotype observed in the *Arabidopsis irx8* mutant, however, was not observed in these *Populus* transgenic RNAi lines. In another study, it was reported that overexpression of full-length *PoGT8D*, the *Populus alba x tremula* homolog of *POPTR_0001s44250/Potri.001G416800* (Phytozome 8.0/Phytozome 10.0), did not complement the *Arabidopsis irx8* mutant [38], although RNAi downregulation of *PoGT8D* suggested a slight reduction in the amount of xylan reducing end sequence [39]. The PoGT8D protein was shown to be targeted to the Golgi, matching its predicted type II membrane topology and in agreement with its involvement in the biosynthesis of non-cellulosic polysaccharides in *Populus* wood. Since the biochemical activity of the GAUT12 protein remains to be determined, it is not clear how this protein is involved in GX biosynthesis in *Populus*. Both of the *GAUT12 Populus trichocarpa* genes are expressed in primary xylem, differentiating xylem, secondary xylem, and phloem fibers in the woody stem. The expression of *PtrGT8D1* has been reported to be seven times greater than *PtrGT8D2* [37,38].

The recalcitrance of *Populus* biomass to deconstruction is the major obstacle to the bioconversion of this lignocellulosic feedstock into biofuels [40]. Heavily acetylated xylan cannot be efficiently hydrolyzed [41] and xylose, xylan, and xylooligomers, when produced during saccharification, inhibit hydrolysis of cellulose by cellulase as well as conversion rates and yields [42]. Reducing xylan levels in *Populus* is, therefore, one strategy to reduce recalcitrance. Similarly, pectin degradation in crop plants as well as in energy plants can enhance saccharification and enable the remaining polymers to be better substrates for the production of biofuels [43-45]. The genetic manipulation of xylan and pectin biosynthetic genes is a potential means to develop *Populus* plants with reduced xylan and pectin content and biomass that yields increased sugar release for biofuel production.

Here, we report that targeted RNAi construct-driven downregulation of one of the *Populus deltoides GAUT12* orthologs (*POPTR_0001s44250/Potri.001G416800*, *GAUT12.1*-KD) results in plants with reduced pectin and xylan content; increased plant height, stem expansion, and biomass yield; and greater glucose and xylose release and lignin syringyl-to-guaiacyl (S/G) ratio. No change in the amount of total lignin was observed in these transgenic lines nor was an irregular xylem cell phenotype observed in secondary *Populus* wood as has been reported in the *Arabidopsis irx8* mutant. There was, however, a significant increase in phloem fiber cell length and width and in xylem fiber and vessel cell size in *PdGAUT12.1* knockdown (*PdGAUT12.1*-KD) wood. *PdGAUT12.1*-KD lines also exhibited larger leaf area, larger palisade, and spongy parenchyma cell size and greater relative water content compared to controls.

## Results and discussion

### *GAUT12* is highly expressed in xylem in *P. deltoides*

**Figure 1:**
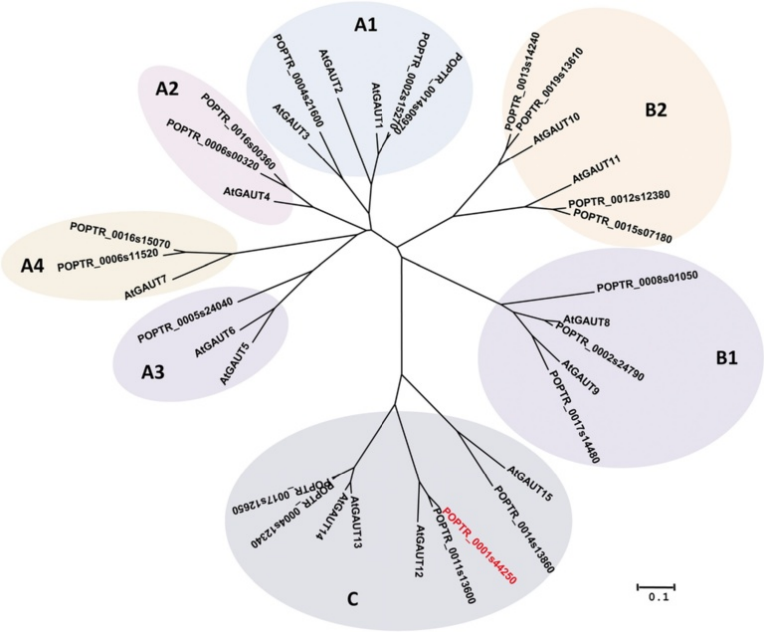
**Phylogenetic tree of GAUTs in** ***Arabidopsis thaliana*** **and** ***Populus trichocarpa*** **.** Phylogenetic tree showing the relationship between amino acid sequences of the GAUT Protein Family of *Arabidopsis thaliana* (TAIR10) and *Populus trichocarpa* (Phytozome 8.0). The tree was constructed by the Neighbor-Joining method using MEGA5 [44]. POPTR_0001s44250 is named *GAUT12.1* in this paper.

**Figure 2:**
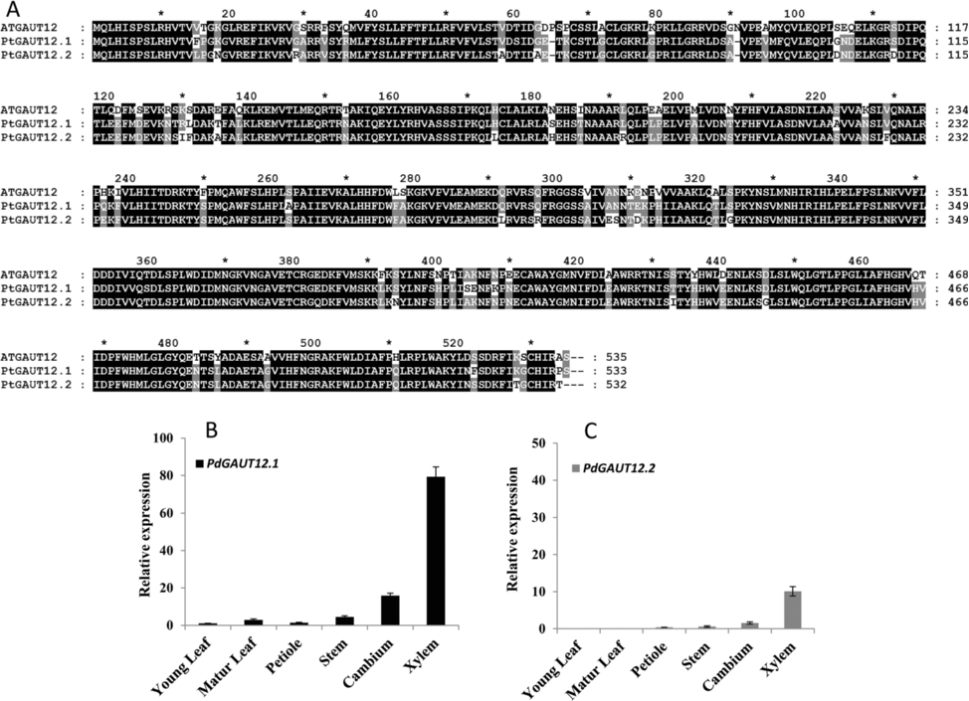
**Sequence alignment of** ***Populus deltoides*** **and Arabidopsis** ***GAUT12*** **homologs and expression of** ***Populus GAUT12*** **genes. (A)** Amino acid sequence alignment between *Arabidopsis* GAUT12 and *Populus* GAUT12. Numbers at the right of each sequence are the positions of amino acid residues in the corresponding proteins. Identical amino acid residues across all three genes are shaded with black and identical residues between two genes are shaded with gray. **(B)** *PdGAUT12.1* and **(C)** *PdGAUT12.2* expression in different tissues of *P. deltoides* as analyzed by quantitative Real-Time PCR. A *Populus 18S ribosomal RNA* (*18S rRNA*) gene was used as internal standard for normalization and the expression of *PdGAUT12.1* in young leaf was set to1. Data are means of three biological replicates ± SE.

The *GAUT1*-related gene family consists of 15 GAUTs in *Arabidopsis* and 20 GAUTs in *P. trichocarpa* [46], suggesting that one or two poplar orthologs exist for each *Arabidopsis GAUT* gene. The *Arabidopsis* GAUT family falls into three broad clades in which clade A is subdivided into clades A1-4, clade B into clades B 1-2, and clade C is undivided [46]. A phylogenetic analysis of the 15 *Arabidopsis* GAUT proteins (TAIR10) and the 20 *P. trichocarpa* GAUTs (Phytozome 8.0) using MEGA5 [47] confirmed the family structure as previously described [46]. There are two *Populus* orthologs of *Arabidopsis* GAUT12 (IRX8) in clade C (Figure 1) [46]. For the purposes of this paper, we named them GAUT12.1 (encoded by POPTR_0001s44250, Genemodel V2.0, Phytozome 8.0; *Potri.001G416800*, Genemodel V3.0, Phytozome 10.0) and GAUT12.2 (encoded by POPTR_0011s13600, Genemodel V2.0, Phytozome 8.0; *Potri.011G132600*, Genemodel V3.0, Phytozome 10.0). PtGAUT12.1 has 82% amino acid identity (Figure 2A) and 75% nucleotide sequence identity to AtGAUT12. The two *Populus* GAUT12 proteins, PtGAUT12.1 and PtGAUT12.2, share 91% amino acid sequence identity and 90% nucleotide sequence identity with each other.

Quantitative real-time (RT)-PCR was used to measure the transcript level of *PdGAUT12.1* and *PdGAUT12.2* in *P. deltoides*. Both genes were most highly expressed in differentiating xylem; however, *PdGAUT12.1* expression was approximately eight times greater than *PdGAUT12.2* (Figure 2B,C) [37,38]. *PdGAUT12.1* transcript expression in leaf, stem, and petiole was lower than in stem cambium in *P. deltoides* (Figure 2B). No *PdGAUT12.2* transcript was detected in leaf and there was low expression in the petiole and stem (Figure 2C). Due to the abundant expression of *PdGAUT12.1* transcript in differentiating *Populus* xylem, we characterized the effect of reduced expression of this gene on plant and wood development and sugar release*.* Several prior reports described aspects of the function of this gene during wood formation in *Populus* [37,39]. However, this is the first report of a knockdown (KD) targeted against the *PdGAUT12.1* gene (*PdGAUT12.1*-KD) alone and of the resulting phenotypes.

### Downregulation of the expression of *GAUT12.1* in *P. deltoides* by RNAi-silencing

**Figure 3:**
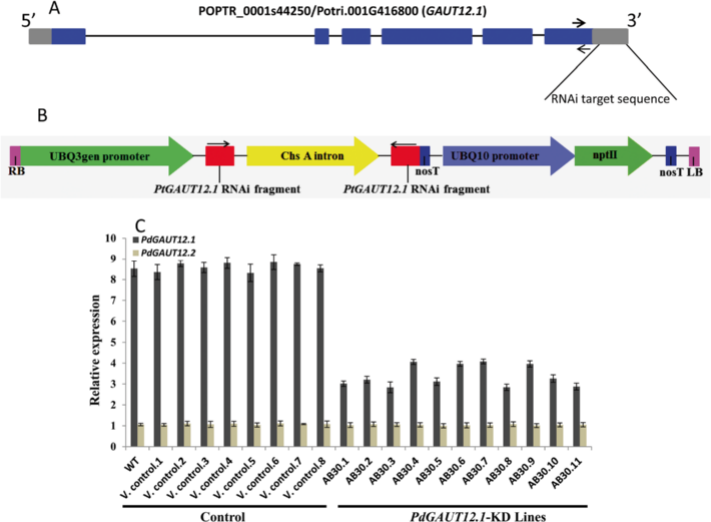
**Knockdown of** ***PdGAUT12.1*** **in** ***Populus deltoides.*** **(A)** Positions of the RNAi target sequence in the *GAUT12.1* (*POPTR_0001s44250/Potri.001G416800*) gene. Blue boxes indicate exons, lines indicate introns, and gray boxes are the 5′ and 3′ untranslated regions (UTRs). The RNAi target sequence was 168 bp in the 3′UTR. The arrows indicate the targeted sequences used for quantitative real-time PCR. **(B)** Schematic presentation of *PdGAUT12.1* RNAi silencing construct. **(C)** Quantitative real-time PCR analysis of controls and *PdGAUT12.1*-KD lines of 3-month-old plants. The relative expression level of each gene was normalized using *Populus 18S ribosomal RNA* (*18S rRNA*) as the reference gene and the expression of *PdGAUT12.2* in AB30.5 was set to 1. *N* = 5. Error bars represent SE.

An RNAi construct (Figure 3B) containing *PtGAUT12.1*-specific sequence to trigger RNAi-silencing of *PdGAUT12.1*, but not *PdGAUT12.2* (Figure 3A), was generated to study the function of GAUT12 during wood development. The construct was transferred into eastern cottonwood (*P. deltoides*) clone WV94. Integration of the *PdGAUT12.1* RNAi construct into the genome was confirmed by gene-specific PCR of leaf DNA from the 11 *PdGAUT12.1*-KD transgenic lines obtained, AB30.1 through AB30.11 (data not shown). The WT non-transformed cottonwood (*P. deltoides*) clone WV94 and eight plants transformed with the vector served as controls. Vector control lines were verified by PCR to contain the control construct (data not shown). Transgenic plants were produced by regeneration of shoots from transformed callus grown on shoot elongation medium, transfer of 3- to 5-cm-tall shoots from callus to BTM medium for rooting [48], and propagation of rooted seedlings to amplify the clonal lines. WT WV94 (25 plants), 10 to 15 clones of each vector control line, and all 11 *PdGAUT12.1*-KD transgenic lines were analyzed for this study.

Transcript expression of *PdGAUT12.1* was measured by quantitative RT-PCR. The transcript level of *PdGAUT12.1* was reduced by 50 to 67% in *GAUT12.1*-KD lines compared to the controls (Figure 3C). Two patterns of *GAUT12.1* transcript reduction were observed. Transgenic lines AB30.1, AB30.2, AB30.3, AB30.5, AB30.8, AB30.10, and AB30.11 had 60 to 67% reduced *GAUT12.1* transcript levels and lines AB30.4, AB30.6, AB30.7, and AB30.9 were reduced by 50 to 54%. We also quantified the transcript level of *GAUT12.2* to determine whether the sequence-specific RNAi construct for *PdGAUT12.1* had any effect on *PdGAUT12.2* transcript expression in the lines. No significant change in *PdGAUT12.2* transcript level was observed in any of the transgenic lines in relation to control plants (Figure 3C).

### RNAi downregulation of *P. deltoides GAUT12.1* expression leads to improved saccharification without changing the amount of total lignin

**Figure 4:**
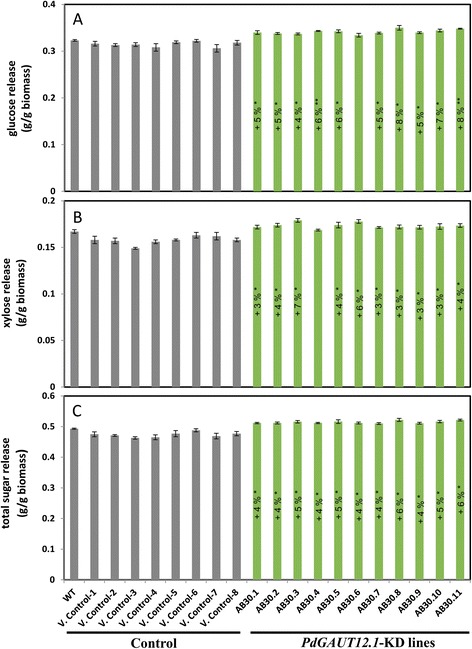
**RNAi downregulation of** ***P. delotides PdGAUT12.1*** **expression leads to improved saccharification.** Glucose **(A)**, xylose **(B)** and total sugar **(C)** release from controls and *PdGAUT12.1*-KD lines. Significance *P* values are expressed as *P < 0.05, **P < 0.001 calculated by statistical analysis using Statistica 5.0 with one-way analysis of variance (ANOVA) followed by Tukey’s multiple comparison test. *n* = 25 for wild-type *P. deltoides*, *n* = 10 to 15 for vector control and *PdGAUT12.1*-KD lines.

To determine the effect of downregulation of *PdGAUT12.1* on the recalcitrance of *Populus* to enzymatic deconstruction, 9-month-old WT, vector control, and *PdGAUT12.1*-KD transgenic lines were subjected to pretreatment and enzymatic hydrolysis. Glucose release per gram dry biomass was significantly increased by 4 to 8% across ten of the eleven *PdGAUT12.1*-KD lines in comparison to the controls (Figure 4A). Xylose release per gram dry biomass was increased 3 to 7% in ten of the eleven transgenic lines (Figure 4B). Total sugar release in all 11 *PdGAUT12.1*-KD lines increased 4 to 6% compared to WT and vector controls (Figure 4C).

*Arabidopsis irx8/gaut12* mutants have been reported to have reduced levels of lignin [30,49]. To determine if the reduced recalcitrance in the *Populus PdGAUT12.1*-KD lines was associated with reduced lignin content, total lignin and lignin S/G levels were measured by pyrolysis molecular beam mass spectrometry. There was no significant difference in the total lignin content of the *PdGAUT12.1*-KD (26.25% dry biomass weight) compared to WT (26.13%) and vector controls (26.19%) (Table 1). This is in contrast to the report on double RNAi-silenced *PtrGT8D1* and *PtGT8D2 P. trichocarpa* lines which had 11 to 25% increased lignin content [37].

**Table 1:** **Total lignin content and S/G ratio of controls and** ***PdGAUT12.1*** **-KD lines**

**Plant types**	**Lignin content (%)**	**S/G ratio**
WT	26.13 ± 0.15	1.90 ± 0.02
V Control-1	25.82 ± 0.17	1.68 ± 0.01
V Control-2	26.68 ± 0.35	1.70 ± 0.03
V Control-3	26.00 ± 0.34	1.62 ± 0.02
V Control-4	26.45 ± 0.21	1.73 ± 0.03
V Control-5	26.43 ± 0.42	1.85 ± 0.03
V Control-6	26.24 ± 0.16	1.85 ± 0.04
V Control-7	25.61 ± 0.40	1.62 ± 0.07
V Control-8	26.30 ± 0.25	1.78 ± 0.05
AB30.1	25.53 ± 0.50	2.00 ± 0.03*
AB30.2	25.99 ± 0.39	1.96 ± 0.02*
AB30.3	26.37 ± 0.15	1.98 ± 0.02 *
AB30.4	26.61 ± 0.26	1.98 ± 0.03*
AB30.5	26.84 ± 0.21	1.97 ± 0.03*
AB30.6	26.11 ± 0.41	2.02 ± 0.04*
AB30.7	26.43 ± 0.38	1.97 ± 0.02*
AB30.8	25.78 ± 0.37	1.97 ± 0.02*
AB30.9	26.44 ± 0.63	2.04 ± 0.02*
AB30.10	26.38 ± 0.24	1.98 ± 0.02*
AB30.11	26.28 ± 0.17	2.03 ± 0.02*

Lignin content is expressed as percentage (wt/wt). S/G ratios were determined by summing the intensity of the syringyl peaks at 154, 167, 168, 182, 194, 208, and 210 and dividing by the sum of intensity of guaiacyl peaks 124, 137, 138, 150, 164, and 178 (see methods for details, Additional file 9). Mean ± SE, Significant *P* values are expressed as *P < 0.05, **P < 0.001 as calculated by statistical analysis using Statistica 5.0 with one-way analysis of variance (ANOVA) followed by Tukey’s multiple comparison test. *n* = 25 for wild-type *P. deltoides*, *n* = 10 to 15 for vector control (v control-1 to v control-8) and *PdGAUT12.1*-KD lines (AB30.1-AB30.11).

All 11 *PdGAUT12.1*-KD lines analyzed showed a significant increase in lignin S/G ratio levels (Table 1), similar to the *Arabidopsis irx8* mutant [30]. There was a positive correlation between S/G ratio and glucose, xylose, and total sugar release (*R*^2^ 0.77, 0.81, and 0.81, respectively). No correlation was observed between sugar release and total lignin content in *PdGAUT12.1*-KD lines. The results indicate that knockdown expression of *GAUT12.1* in *P. deltoides* results in increased sugar release and lignin S/G ratios.

A small reduction in S lignin was previously reported in *P. alba x tremula PoGT8D* RNAi lines [39]. However, in the present study with *P. deltoides* the S/G ratio was significantly increased (3 to 7%) in the *PdGAUT12.1*-KD lines compared to WT and vector control plants (Table 1). Total sugar release was previously shown to increase with increasing S/G ratio [50]. However, in that study, glucose and total sugar release were negatively correlated with lignin content. In the present study, although total lignin levels did not differ between the control and transgenic lines, there was still a positive correlation between high S/G ratios and increased sugar release in the *PdGAUT12.1*-KD lines, suggesting that *GAUT12.1* expression affects the lignin composition in wood and that the composition of the lignin is a factor affecting recalcitrance. It is also noteworthy that, contrary to the results obtained here, simultaneous knockdown expression of both *Populus GAUT12* homologs in *P. trichocarpa* resulted in 11 to 25% increased lignin content [37]. As there was no measurement of biomass recalcitrance in that report, it is not known how the transgenic plants were affected in sugar release. Other studies, however, have shown that increased lignin content is detrimental to effective sugar release [50-52]. The knockdown of both *GAUT12* homologs in *P. trichocarpa* transgenics also resulted in more brittle wood, 17 to 29% reduced stem modulus of elasticity and 16 to 23% reduced modulus of rupture [37], suggesting that reduced expression of both homologs may be deleterious to wood quality.

### RNAi downregulation of *PdGAUT12.1* results in increased plant growth in Poplar

**Figure 5:**
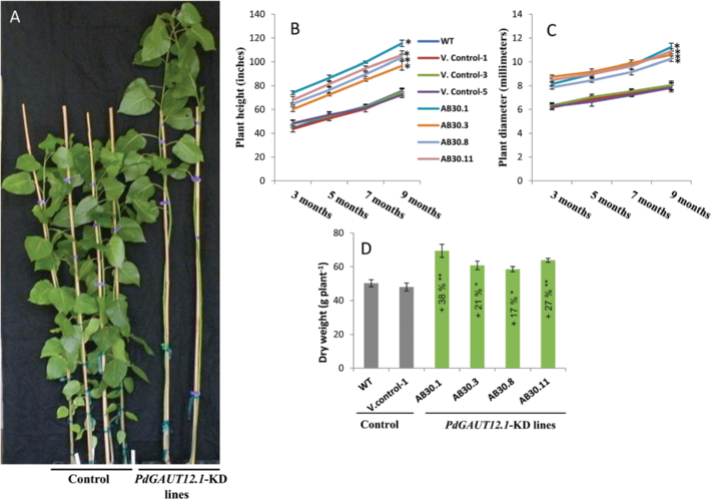
**RNAi downregulation of** ***PdGAUT12.1*** **results in increased plant growth.** Phenotype and development of *PdGAUT12.1*-KD transgenic lines. **(A)** Plant phenotypes of 3-month-old wild-type *P. deltoides* (left two plants), vector control (middle two plants), and *PdGAUT12.1*-KD lines (right two plants). **(B)** Height and **(C)** radial growth of *PdGAUT12.1*-KD lines in comparison to the controls. **(D)** Dry aerial biomass of WT and *PdGAUT12.1*-KD lines of three-month-old plants. *n* = 7 for plant height and stem diameter, *n* = 6 for dry weight measurement. Asterisks indicate values significantly different from WT by ANOVA followed by Tukey’s multiple comparison test, *P < 0.05, **P < 0.001. Error bars represent SE.

Substantial morphological differences were observed between the controls and *PdGAUT12.1*-KD transgenic lines. Measurement of plant height and stem diameter at 3 months showed that *PdGAUT12.1* RNAi-silenced transgenic lines had increased vegetative growth compared to controls (Figure 5A). Nine of the eleven *PdGAUT12.1*-KD transgenic lines had 12 to 52% increased plant height compared to WT and vector control plants (Table 2), and nine lines had 12 to 44% greater stem diameter (Table 2). We selected four transgenic lines (AB30.1, AB30.3, AB30.8, AB30.11) for further phenotype analysis based on GAUT12.1 transcript expression and growth phenotype, lignin S/G ratio, and the saccharification data. Measurement of plant height and stem diameter over a 9-month growth period showed that the increased height and stem diameter of the *PdGAUT12.1*-KD transgenic lines continued throughout the growth period (Figure 5B,C). Indeed, the total aerial biomass of the *PdGAUT12.1*-KD plants was 17% to 38% greater than controls (Figure 5D) by the end of the 9-month growth period. The increase in plant height and stem diameter were negatively correlated (*R*^2^ of 0.66 and 0.75, respectively) with *GAUT12.1* transcript levels (Additional file 1), which were reduced by 50 to 67% compared to WT and vector control plants. These results suggest that systematic manipulation of the *GAUT12.1* gene can lead to greater plant growth.

**Table 2:** **Height and diameter of** ***PdGAUT12.1*** **-KD and control lines**

**Plant types**	**Plant height (inches)**	**Plant diameter (mm)**
WT	48.31 ± 3.1	6.3 ± 0.24
V Control-1	46.67 ± 2.5	6.1 ± 0.19
V Control-2	47.18 ± 0.9	6.2 ± 0.13
V Control-3	47.95 ± 1.2	6.3 ± 0.26
V Control-4	47.62 ± 1.9	6.1 ± 0.31
V Control-5	48.43 ± 2.0	6.3 ± 0.37
V Control-6	48.51 ± 1.5	6.1 ± 0.36
V Control-7	48.81 ± 2.0	6.3 ± 0.22
V Control-8	48.81 ± 1.0	6.1 ± 0.23
AB30.1	74.17 ± 2.9**	8.2 ± 0.46**
AB30.2	61.81 ± 3.3**	7.7 ± 0.34**
AB30.3	60.29 ± 2.3**	8.7 ± 0.32**
AB30.4	49.22 ± 2.2	6.8 ± 0.29
AB30.5	60.86 ± 2.0**	9.1 ± 0.53**
AB30.6	54.61 ± 3.8**	6.6 ± 0.41
AB30.7	49.86 ± 2.4	7.1 ± 0.28**
AB30.8	64.75 ± 2.4**	7.9 ± 0.37**
AB30.9	54.82 ± 1.0**	7.2 ± 0.37**
AB30.10	61.72 ± 2.3**	8.4 ± 0.30**
AB30.11	68.07 ± 2.0**	8.5 ± 0.33**

Plant height and plant diameter of three-month-old wild type *P. deltoides*, vector control and *PdGAUT12.1*-KD lines. Means ± SE, Significance *P* values are expressed as **P < 0.05, **P < 0.001 as calculated by statistical analysis using Statistica 5.0 with one-way analysis of variance (ANOVA) followed by Tukey’s multiple comparison test. n = 25 for wild type *P. deltoides*, n = 10 to 15 for vector control (v control-1 to v control-8) and *PdGAUT12.1*-KD lines (AB30.1-AB30.11).

The *PdGAUT12.1*-KD plants had increased vegetative growth compared to controls. This is in contrast to *Arabidopsis irx8* mutant plants which have stunted growth [8,10,29]. This difference may be due to the 50 to 67% reduction in *GAUT12.1* transcript level in the RNAi-silenced *P. deltoides* transgenic plants compared to the total knock out of *GAUT12/IRX8* in the *Arabidopsis irx8* mutants [8,29]. However, the previously reported simultaneous knockdown expression of both *PtrGT8D1* and *PtGT8D2* in *P. trichocarpa*, similar to the work described here, also did not yield a dwarf phenotype in *Populus* even though those plants had 85 to 94% reduced *GAUT12* transcript levels [37]. The *PtrGT8D* RNAi-silenced transgenic plants [37] also did not have collapsed vessels as observed in the *Arabidopsis irx8* mutant [8,29]. Based on those results, it was proposed that growth of the *PtrGT8D* RNAi-silenced transgenic plants was not affected because the vessels in the transgenic lines retained their normal transport function [37]. The simultaneous knockdown expression of both *Populus GAUT12* homologs in *P. trichocarpa* [33], however, did not result in the increased growth observed in the current study where only *GAUT12.1* expression was reduced. The increased plant size obtained upon targeted downregulation of a specific *GAUT12* homolog as reported here suggests that a controlled manipulation of this gene to yield greater plant growth could be used to improve feedstock for the biofuel industry.

### Altered *PdGAUT12.1* expression in leaves causes increased leaf cell size and number and increased relative water content

To determine how leaf growth and size was affected in the *PdGAUT12.1*-KD lines, every fifth successive leaf from the apex down to leaf 40 (Additional file 2C-D) was examined. Both leaf length and width was significantly (*P* = 0.05) larger for each of the *PdGAUT12.1*-KD lines examined compared to the controls (Additional file 2C-D). To compare the growth of developing leaves, every fifth, sixth, and seventh leaf from the apex was collected (Additional file 2B, E) and leaf area was measured. All *PdGAUT12.1*-KD lines examined had 52 to 117% significantly greater leaf area compared to control plants (Additional file 2E). Simultaneously, we collected the 20th to 40th leaf from the apex of five transgenic and control lines and compared the fully expanded leaf area. All four transgenic lines had 22 to 76% greater leaf area than control plants (Additional file 2 F). Therefore, both the leaf length and width was significantly greater in both the developing and expanded leaves of *PdGAUT12.1*-KD lines resulting in a larger leaf area. The *PdGAUT12.1*-KD lines also had accelerated growth in the top part of the *PdGAUT12.1*-KD plant (Additional file 2A) and 2.8 times greater biomass yield compared to WT (Additional file 2H).

To establish whether the larger leaf size in the *PdGAUT12.1*-KD lines was a result of increased cell number or cell expansion, we analyzed cross sections of leaf blades of the sixth leaf from the apex of the plant and hand cut cross sections of the 20th leaf. The knockdown lines had increased numbers of palisade parenchyma cells (17%, AB30.1; 13%, AB30.8) compared to WT (Additional file 3A-C, M). The knockdown lines also had larger bundle-sheath cells (Additional file 3D-G, I, K). Although there were more palisade cells in the young leaves, by the time these leaves matured, there was no overall major change in cross sectional total leaf area occupied by palisade cells of *PdGAUT12.1*-KD lines compared to WT, except for a small 9% increase in line AB30.1 (Additional file 4A-F). As shown in Additional file 2; however, there was a significant increase in the total leaf surface area in the mature *PdGAUT12.1*-KD leaves compared to the control (Additional file 2C-F). Also, the palisade and spongy parenchyma cells in mature leaves of *PdGAUT12.1*-KD lines were 13 to 56% and 23 to 62% larger, respectively, than in the controls (Additional file 4G-H). Thus, the increased leaf size of the *PdGAUT12.1*-KD lines is due to both increased cell number and cell size.

Leaf water content is a useful indicator of plant water balance and expresses the relative amount of water present in the plant tissues. The measurement of relative water content (RWC) in leaf tissues is commonly used to assess the water status of plants. Measurements of water content in a tissue on a fresh or dry mass basis can be correlated with the maximum amount of water a tissue can hold. Leaf water status is positively correlated with several leaf physiological variables, such as leaf turgor, transpiration, stomatal conductance, and growth [53]. We thus measured the RWC of *PdGAUT12.1*-KD lines to determine if it was correlated with the positive growth effect observed in the lines. All four knockdown lines examined had 5 to 10% increased RWC compared to the WT at 72 h (Additional file 2G). After 72 h, there was a 7% increase in RWC of *PdGAUT12.1*-KD plants compared to the WT (Additional file 2G). In summary, the results show that the increased leaf size in the *PdGAUT12.1*-KD lines could be explained by enhanced cell number, enhanced cell size, and enhanced RWC.

### Downregulation of *PdGAUT12.1* in secondary cell wall-containing tissues leads to increased phloem and vessel size

**Figure 6:**
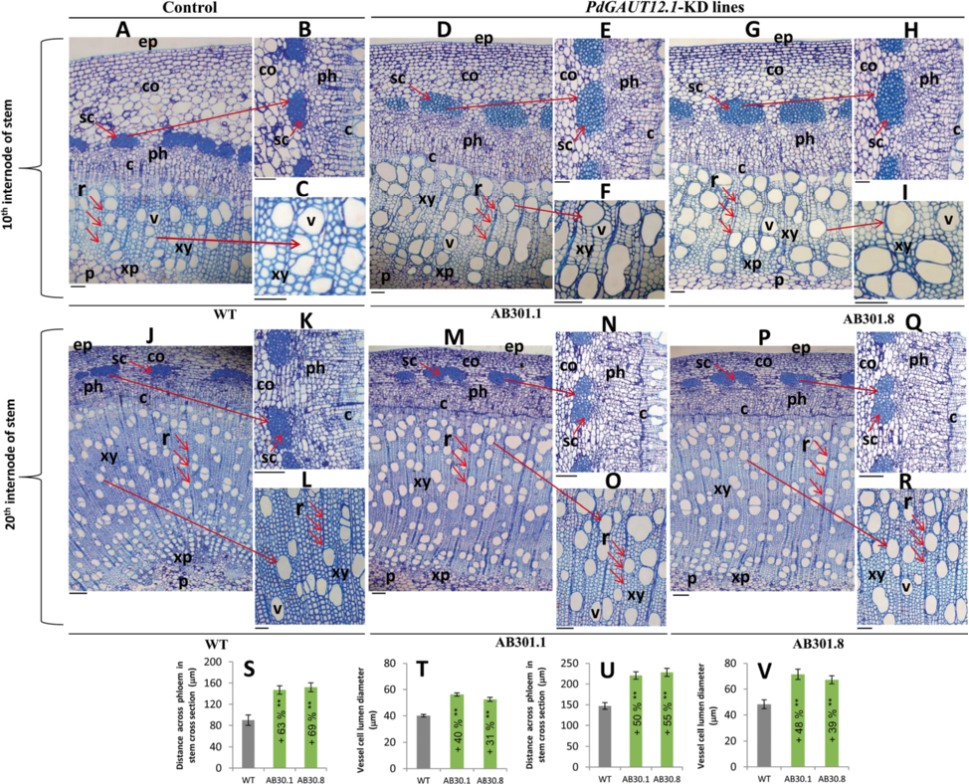
**RNAi downregulation of** ***P. deltoides PdGAUT12.1*** **expression leads to increased phloem and vessel size.** Cross sections (1 μm) of three-month-old stems of wild-type WV-94 (A-C, J-L) and *PdGAUT12.1*-KD transgenic lines AB30.1 (D-F, M-O) and AB30.8 (G-I, P-R) stained with toluidine blue. **A** Cross section of the 10th internode of stem from a wild type WV-94 plant. **B**, **C** Higher magnification of Figure A. **J** cross section of 20th internode of a WT stem. **K, L** Higher magnification of Figure J. **D** Cross section of the 10th internode of a stem from transgenic line AB30.1. **E, F** Higher magnification of Figure D. **M** Cross section of 20th internode of stem from AB30.1. **N, O** Higher magnification of Figure M. **G** Cross section of the 10th internode of a stem from AB30.8. **H, I** Higher magnification of Figure G. **P** Cross section of the 20th internode of stem from AB30.8. **Q, R** Higher magnification of Figure P. **S,T** Distance across phloem in stem cross section (S) and xylem vessel cell lumen diameter size (T) from A, D, and G of the 10th internode from wild type and transgenic lines. **U, V** Distance across phloem in stem cross section (U) and xylem vessel cell lumen diameter size (V) from J, M, and P of 20th internode of 20 WT and transgenic lines. Red arrows show ray cells and the area of cross sections exposed to higher magnification. ep: epidermis, co: cortex, sc: sclerenchyma fibers ph: phloem, c: cambium, xy: xylem, r: xylem ray cells, xp: xylem parenchyma, v: xylem vessel, p: pith. Bar = 50 μm, A to C, D, E, G, H, L; 100 μm, J, K, M, N, P, Q; 70 μm, F, I, O, R. Error bars represent SE. *P < 0.05, **P < 0.001.

To examine the role of downregulation of *GAUT12.1* in tissues containing secondary walls, stems from transgenic lines and WT were analyzed by sectioning internode numbers 10 and 20 from the top of the stem. A comparison of the internode 10 stem cross sections from *PdGAUT12.1*-KD lines versus WT revealed a significant increase in the *PdGAUT12.1*-KD lines of the amount of phloem tissue (63%, AB30.1; 69%, AB30.8) compared to WT (Figure 6A,B,D,E,G,H,S). We did not observe the collapsed xylem cell phenotype reported for the *irx8 Arabidopsis* mutants [8,29]. However, there was a significant increase in xylem vessel cell size in the *PdGAUT12.1*-KD lines compared to WT. The 10th internode from the transgenic lines had 40% and 31% larger vessel diameter than WT (Figure 6A,C,D,F,G,I,T). Our data corroborate similar vessel phenotypes previously reported in *P. trichocarpa* when both *PtGAUT12.1* and *PtGAUT12.2* were simultaneously knocked down through RNAi silencing [37]. We also observed an increased amount of phloem (50%, AB30.1; 55%, AB30.8) and increased xylem vessel cell lumen diameter (48%, AB30.1; 39%, AB30.8) in the 20th internodes from the transgenic lines compared to the WT (Figure 6J to R,U,V).

**Figure 7:**
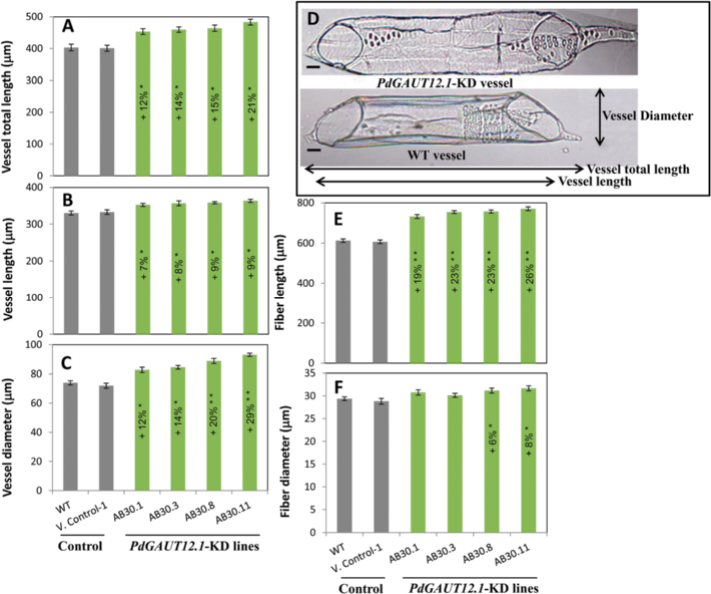
**Xylem (fiber and vessel) cell size in WT and** ***PdGAUT12.1*** **-KD line.** Downregulation of expression of *GAUT12.1* results in increased xylem total vessel length **(A)**, xylem lumen length **(B)**, xylem vessel lumen diameter **(C)**, xylem fiber cell length **(E)**, xylem fiber cell diameter **(F)**. Both xylem fibers and vessels were separated by maceration and approximately 80 fiber cells and 60 vessel elements from three independent *PdGAUT12.1*-KD transgenic lines were analyzed. Images were captured with a Nikon DS-Ri1 camera (Nikon, Melville, NY) using NIS-Elements Basic Research software. **(D)** Representative individual vessels of WT and *PdGAUT12.1*-KD lines illustrating the parameters measured. Asterisks indicate values significantly different from wild type based on ANOVA followed by Tukey’s multiple comparison test, *P < 0.05, **P < 0.001. *n* = 240 fibers and 180 vessels cells. The vertical bar represents standard error. Bar = 20 μm, D.

Since we observed increased stem diameter in the *PdGAUT12.1*-KD lines, we hypothesized that increased wood diameter was a result of an increased radial expansion of the xylem cells. To test this hypothesis, wood cells from internode 40 of the transgenic and WT plants were separated by maceration. The measurement of fiber cell size revealed that all knockdown lines had greater fiber cell length (19% to 26%) and two transgenic lines had wider fiber cells (6%, 8%) than the WT (Figure 7E,F). Vessel cell length and diameter were also measured in the WT and *PdGAUT12.1*-KD lines (Figure 7D). All transgenic lines examined had significantly larger vessel cell total length (12% to 21%) and vessel cell lumen length (7% to 9%) and wider vessel cell diameter (12% to 29%) than WT vessels (Figure 7A to C). Thus, at least a portion of the change in stem length and diameter in *PdGAUT12.1*-KD lines is likely due to increased xylem and vessel cell size. The larger vessel size may also support the larger leaf size and greater water content of the *pdGAUT12*-KD lines.

### *Populus PdGAUT12.1* complements and *PdGAUT12.2* partially complements the Arabidopsis *irx8* (*gaut12*) mutant

**Figure 8:**
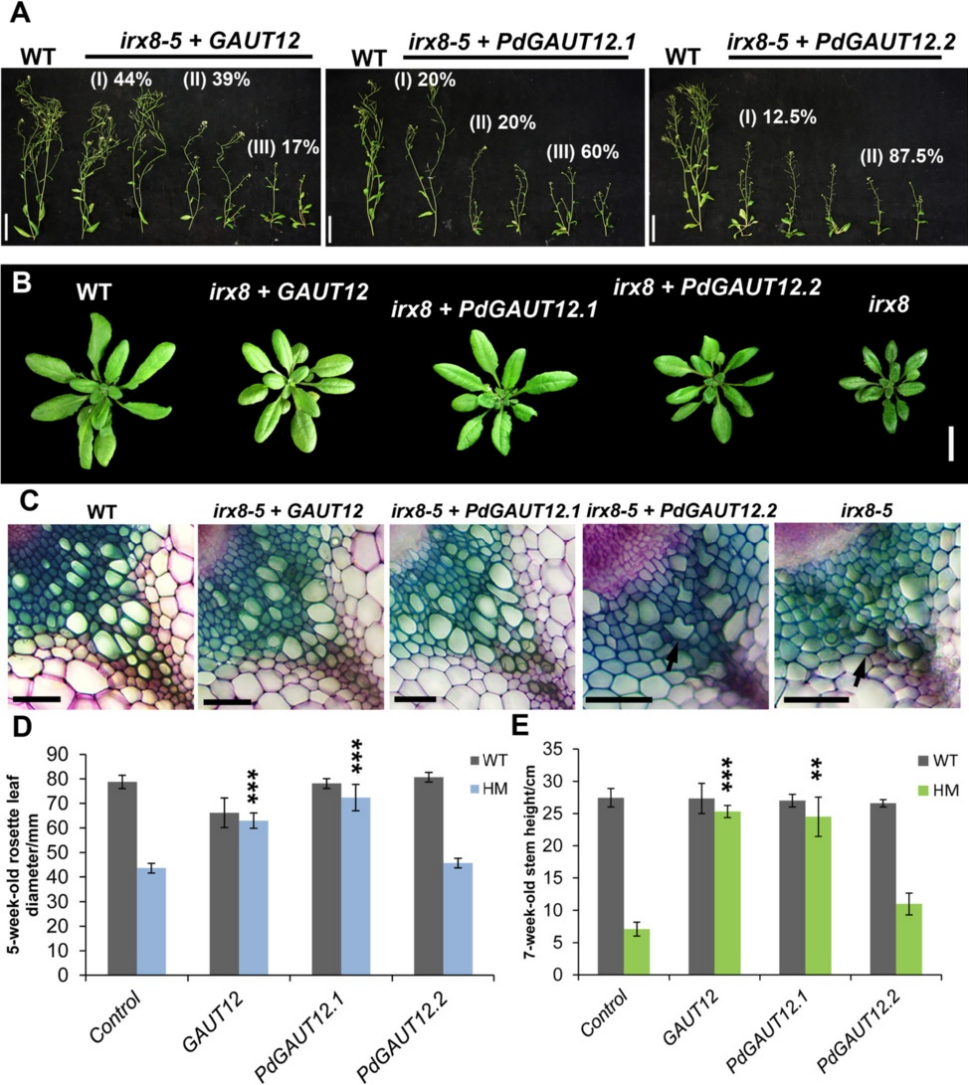
**Characterization of** ***irx8*** **mutants complemented by CaMV35S driven** ***GAUT12*** **,** ***PdGAUT12.1*** **, and** ***PdGAUT12.2*** **.** **(A)** Pictures of 7-week-old wild-type (WT), and *irx8 + GAUT12*, *irx8 + PdGAUT12.1*, and *irx8 + PdGAUT12.2* complemented lines. The complemented lines were classified into groups according to the phenotypes and labeled by percentage. Bar = 5 cm. **(B)** Images of 5-week-old rosette leaves of WT, *irx8 + GAUT12*, *irx8 + PdGAUT12.1*, *irx8 + PdGAUT12.2*, and *irx8*. Bar = 2 cm. **(C)** Hand-cut transverse sections of the basal stems stained with Toluidine blue O, arrows indicate collapsed xylem vessels. Bar = 50 μm. Measurements of **(D)** 5-week-old rosette leaf diameter and **(E)** 7-week-old stem height. Value = mean ± standard error (*n* ≥ 3), Value = mean ± standard deviation (n = 3), ***P < 0.001, significant increase of rosette leaf size or stem height compared to the *irx8* mutant determined by one-way ANOVA and *post hoc* Bonferroni corrected *t*-test.

To examine whether *PdGAUT12.1* and *PdGAUT12.2* are functional homologs of the Arabidopsis *GAUT12*, we transformed T-DNA constructs harboring the full-length coding sequences of *Arabidopsis thaliana GAUT12* as well as *P. deltoides GAUT12.1* and *GAUT12.2* (Additional file 5) under the control of a CaMV 35S promoter into *irx8-5/+* plants. For each construct, approximately 12 primary transgenics homozygous for the *irx8* allele were selected and their phenotypes examined. Both the *AtGAUT12* and *PdGAUT12.1* constructs showed similar activity in complementing *irx8*: about 20% of the primary transgenics did not show any of the visible defects associated with the mutation; these plants produced WT-like rosette leaves and intact vessels (Figure 8A to C), resulting in significantly increased rosette size and overall height (Figure 8D,E). In contrast, the *PdGAUT12.2* construct only partially rescued the phenotypes associated with *irx8*, as primary transgenics had only marginally larger rosette leaves and slightly taller stature (Figure 8D,E). The *PdGAUT12.2* lines displaying the strongest rescue effect still had many collapsed xylem vessels (Figure 8C). We conclude that the Poplar *PdGAUT12.1* gene is as effective in complementing Arabidopsis *irx8* mutants as the native *GAUT12* gene.

### RNAi downregulation of *GAUT12.1* expression leads to reductions in pectin and xylan content and increased extractability of *P. deltoides* wood

#### Sugar composition analyses of total cell walls from *PdGAUT12.1*-KD plants

We investigated the effects of RNAi downregulation of *P. deltoides GAUT12.1* expression on the content of cell wall polysaccharides in *P. deltoides* wood. Cell walls were isolated as alcohol-insoluble residues (AIRs) from cored, debarked wood from the bottom 6 cm of stems. All four *PdGAUT12.1*-KD lines had 17 to 30% significantly reduced xylose content in AIR compared to control lines (Table 3), indicating that RNAi downregulation of *P. deltoides GAUT12.1* expression causes a defect in xylan formation. This result corroborated two earlier findings in *Populus* that *PtGAUT12.1* has a role in xylan formation [37,39]. Simultaneously, the galacturonic acid (GalA) content was significantly reduced by 25% to 47% in all four transgenic lines compared to the WT. This result indicates that *PdGAUT12.1*-KD lines have defects in pectin formation in addition to the defect in xylan formation. A substantial increase in galactose and mannose content was also observed in *PdGAUT12.1*-KD walls compared to controls. The AIR composition data indicated a clear alteration in the composition of the *PdGAUT12.1*-KD walls compared to controls and indicated that at least two types of polymers, xylan and pectin, were affected.

**Table 3:** **Monosaccharide sugar composition of total cell walls (AIR) from nine-month-old** ***Populus*** **plants**

	**Sugar composition of wood AIR stems walls (mol%; ± SE standard error)**								
	**Ara**	**Rha**	**Fuc**	**Xyl**	**GlcA**	**GalA**	**Man**	**Gal**	**Glc**
WT	3.11 ± 0.11	2.01 ± 0.05	0.08 ± 0.01	46.27 ± 1.02	0.23 ± 0.01	6.31 ± 0.13	6.29 ± 0.41	3.07 ± 0.32	33.21 ± 0.85
V Control-1	3.00 ± 0.12	1.87 ± 0.11	0.09 ± 0.01	47.01 ± 1.11	0.22 ± 0.02	6.35 ± 0.11	6.61 ± 0.42	3.11 ± 0.47	32.25 ± 0.99
AB30.1	3.01 ± 0.09	2.31 ± 0.12	0.10 ± 0.01	38.33 ± 0.56*	0.21 ± 0.02	4.70 ± 0.14*	10.09 ± 0.36*	7.32 ± 0.33*	33.82 ± 0.76
AB30.3	2.95 ± 0.08	2.01 ± 0.13	0.06 ± 0.01	36.14 ± 1.01*	0.21 ± 0.01	4.30 ± 0.09*	11.58 ± 0.38*	8.53 ± 0.53*	34.19 ± 1.01
AB30.8	2.76 ± 0.01	1.76 ± 0.21	0.04 ± 0.01	32.30 ± 0.85**	0.20 ± 0.01	3.34 ± 0.08**	14.59 ± 0.53*	10.99 ± 0.58*	33.98 ± 0.89
AB30.11	2.86 ± 0.08	1.85 ± 0.07	0.17 ± 0.02	34.44 ± 1.12**	0.19 ± 0.01	3.79 ± 0.11**	11.72 ± 0.39*	10.95 ± 0.61*	33.96 ± 0.97

Glycosyl residue composition of stem AIR as measured by GC-MS of tetramethylsilane (TMS) derivatives from controls and *PdGAUT12.1*-KD lines. The amount of sugar is represented as average mol% of AIR (alcohol insoluble residue) isolated from stem walls. Means ± SE of three biological replicates with two technical replicates each. Ital values with asterisks represent significant difference between WT and *PdGAUT12.1*-KD lines at *P ≤ 0.05, **P ≤ 0.001 significant level (one-way ANOVA followed by Tukey’s multiple comparison test).

#### Glycosyl residue composition analyses of fractionated cell walls from *PdGAUT12.1*-KD plants

The AIR (that is, cell walls) of control and transgenic lines were subjected to sequential extraction with increasingly harsh solvents to fractionate the walls to determine if cell wall changes caused by the *GAUT12.1*-KD could be identified in a specific subfraction of cell wall polymers. The total amount of material recovered in each sequential extract of the starting AIR for each of the six solvents was greater for the *PdGAUT12.1*-KD RNAi lines than for the controls. There was a significant 9 to 21% increase in the amount of material recovered in the ammonium oxalate extract, 7 to 15% increase in the carbonate extract, 17 to 34% increase in the 1 M KOH extract, 12 to 30% increase in the 4 M KOH extract, 8 to 10% increase in the chlorite extract, and 7 to 15% increase in the post-chlorite 4 M KOH extract, yielding an 11 to 17% overall increase in the total amount of extractable material from the starting AIR of the *PdGAUT12.1*-KD lines compared to WT (Additional file 6A-H). Since the total amount of AIR isolated from the WT versus the transgenic lines was comparable (Additional file 6A), these results suggested that the remaining insoluble wall material should have been reduced in the transgenic lines. Indeed, analysis of the mass of the final AIR pellet remaining after all extractions of AIR from the transgenic lines revealed that there was 6 to 13% less final pellet from the *PdGAUT12.1*-KD lines compared to control lines (Additional file 6I). These results suggest that GAUT12 functions in the synthesis of a structure that is highly integrated into the basic cell wall architecture, and that when GAUT12 function is reduced, the plants synthesize more easily extractable walls.

To determine if there were any differences in the type or mol% amounts of each type of sugar in the different extracts from the *PdGAUT12.1*-KD lines compared to controls, the individual extracts were analyzed by glycosyl residue composition. Wall fractions extracted with mild reagents such as ammonium oxalate and sodium carbonate generally contain large amounts of GalA along with rhamnose (Rha), arabinose (Ara), galactose (Gal), glucose (Glc), xylose (Xyl), and mannose (Man) and, thus, are considered to be enriched in pectin. The oxalate extracts of AIR from the *PdGAUT12.1*-KD RNAi line had 39 to 78% decreased mol% GalA compared to the controls, and the carbonate extracts had 17 to 42% decreased mol% GalA (Table 4). The mol% rhamnose in both the oxalate and carbonate fractions was also significantly reduced by 44 to 76% and 39 to 55%, respectively, in the *PdGAUT12.1*-KD lines compared to the controls (Table 4). These results show that *GAUT12.1* downregulation results in reduced amounts of easily extractable HG and RG. Simultaneously, the xylose, mannose, galactose, and glucose contents in the oxalate extracts were significantly increased in the *PdGAUT12.1*-KD lines compared to the WT (Table 4), while in the carbonate fractions, the glucose content was increased, suggesting that reduced levels of easily extractable pectin were accompanied by increased mol% hemicellulose in these wall fractions.

**Table 4:** **Glycosyl residue composition of cell wall (AIR) fractions from stem of controls and** ***PdGAUT12.1*** **-KD plants**

		**Sugar composition of cell wall fractions (mol %; ± SE standard error)**							
	**Ara**	**Rha**	**Fuc**	**Xyl**	**GlcA**	**GalA**	**Man**	**Gal**	**Glc**
Ammonium oxalate									
WT	20.9 ± 1.0	4.1 ± 0.2	0.7 ± 0.02	8.9 ± 0.2	0.6 ± 0.01	23.1 ± 1.0	11.4 ± 0.9	3.7 ± 0.3	26.8 ± 1.2
V Control-1	21.0 ± 0.6	4.9 ± 0.3	0.8 ± 0.02	8.3 ± 0.4	0.5 ± 0.02	23.2 ± 1.1	11.6 ± 1.0	3.6 ± 0.2	26.9 ± 1.0
AB30.1	18.5 ± 0.4	1.0 ± 0.3**	0.2 ± 0.01	10.2 ± 0.5	0.7 ± 0.02	14.2 ± 1.3*	19.5 ± 1.1*	5.6 ± 0.3*	30.6 ± 0.9*
AB30.3	17.7 ± 0.6*	2.1 ± 0.2*	0.4 ± 0.01	10.5 ± 0.2*	0.7 ± 0.01	12.9 ± 0.9**	18.2 ± 1.3*	6.0 ± 0.1*	31.4 ± 1.0*
AB30.8	19.8 ± 0.8	2.1 ± 0.1*	0.4 ± 0.01	11.1 ± 0.3*	0.8 ± 0.01	5.0 ± 0.5**	21.9 ± 1.2*	6.2 ± 0.3*	32.8 ± 1.1*
AB30.11	19.3 ± 0.6	2.3 ± 0.2*	0.4 ± 0.02	11.4 ± 0.3*	0.8 ± 0.01	8.9 ± 0.8**	20.2 ± 1.3*	7.1 ± 0.2*	*29.4 ± 1.2*
Sodium carbonate									
WT	10.7 ± 0.6	6.0 ± 0.2	0.3 ± 0.02	23.0 ± 0.2	1.6 ± 0.01	19.3 ± 0.5	14.0 ± 0.9	7.0 ± 0.3	18.0 ± 0.9
V Control-1	11.1 ± 0.4	5.9 ± 0.1	0.5 ± 0.02	22.5 ± 0.6	1.5 ± 0.02	19.5 ± 0.8	15.0 ± 0.8	6.7 ± 0.2	17.9 ± 0.7
AB30.1	4.8 ± 0.4*	2.7 ± 0.2*	0.4 ± 0.01	24.2 ± 0.5	1.7 ± 0.02	16.0 ± 0.3*	18.0 ± 1.0	5.8 ± 0.3	26.9 ± 0.9*
AB30.3	7.3 ± 0.3	2.7 ± 0.1*	0.3 ± 0.01	24.7 ± 0.2	1.7 ± 0.01	14.0 ± 0.4*	17.2 ± 0.6	6.8 ± 0.3	25.0 ± 1.0*
AB30.8	7.9 ± 0.3	3.7 ± 0.2*	0.8 ± 0.01	26.2 ± 0.3	2.6 ± 0.01	11.2 ± 0.5*	19.3 ± 0.9	6.7 ± 0.4	21.9 ± 1.1
AB30.11	7.3 ± 0.4	3.4 ± 0.3*	0.4 ± 0.02	25.7 ± 0.3	1.9 ± 0.01	13.5 ± 0.4*	16.3 ± 0.7	7.4 ± 0.2	24.2 ± 1.2*
1 M KOH									
WT	0.5 ± 0.04	4.2 ± 0.2	0.2 ± 0.02	69.3 ± 1.9	8.5 ± 0.3	3.3 ± 0.1	6.4 ± 0.6	1.7 ± 0.2	7.4 ± 1.2
V Control-1	0.6 ± 0.03	4.1 ± 0.1	0.3 ± 0.01	70.1 ± 2.3	8.7 ± 0.2	3.4 ± 0.2	6.0 ± 0.5	1.6 ± 0.3	7.1 ± 1.4
AB30.1	1.1 ± 0.05	3.1 ± 0.2	0.4 ± 0.01	44.8 ± 2.3*	5.5 ± 0.2*	1.3 ± 0.09*	10.7 ± 0.5*	4.1 ± 0.3**	29.3 ± 2.1**
AB30.3	1.6 ± 0.06	3.1 ± 0.1	0.4 ± 0.01	42.4 ± 2.4*	5.3 ± 0.3*	1.2 ± 0.1*	11.1 ± 1.0*	3.7 ± 0.1**	31.9 ± 2.5**
AB30.8	1.8 ± 0.1	2.6 ± 0.1*	0.5 ± 0.01	37.8 ± 2.6**	4.7 ± 0.4*	0.8 ± 0.08**	14.6 ± 0.8*	4.8 ± 0.2**	33.2 ± 2.1**
AB30.11	1.6 ± 0.06	3.3 ± 0.2	0.4 ± 0.02	38.4 ± 3.2**	4.8 ± 0.3*	1.0 ± 0.1**	11.2 ± 0.4*	3.8 ± 0.2**	35.9 ± 2.4**
4 M KOH									
WT	2.3 ± 0.08	3.0 ± 0.1	0.5 ± 0.02	37.8 ± 1.3	4.7 ± 0.2	0.8 ± 0.04	11.9 ± 1.2	7.8 ± 0.3	31.2 ± 1.2
V Control-1	2.4 ± 0.06	3.2 ± 0.2	0.3 ± 0.01	38.1 ± 1.5	4.9 ± 0.2	0.9 ± 0.03	11.4 ± 0.5	7.9 ± 0.3	30.8 ± 1.5
AB30.1	1.6 ± 0.09	2.4 ± 0.1	0.4 ± 0.01	26.2 ± 1.7*	3.4 ± 0.2*	0.5 ± 0.03*	18.2 ± 0.5**	*8.4 ± 0.3*	38.9 ± 1.8*
AB30.3	2.1 ± 0.07	2.2 ± 0.2	0.3 ± 0.01	25.2 ± 1.1*	3.3 ± 0.3*	0.4 ± 0.02*	15.6 ± 1.0*	10.9 ± 0.5**	39.9 ± 1.5*
AB30.8	1.7 ± 0.05	1.9 ± 0.2*	0.3 ± 0.01	22.3 ± 1.3**	2.7 ± 0.4*	0.3 ± 0.03**	18.9 ± 0.8**	8.6 ± 0.6*	43.2 ± 1.3**
AB30.11	1.9 ± 0.09	2.3 ± 0.1	0.4 ± 0.02	23.2 ± 1.4**	2.9 ± 0.3*	0.3 ± 0.05**	15.9 ± 0.4*	*8.1 ± 0.2*	44.9 ± 1.9**
Chlorite									
WT	7.2 ± 0.5	4.3 ± 0.1	-	10.9 ± 0.5	0	6.4 ± 0.2	2.1 ± 0.1	11.2 ± 0.4	57.9 ± 1.1
V Control-1	7.0 ± 0.3	4.4 ± 0.1	0.2 ± 0.02	11.1 ± 0.7	0	6.5 ± 0.3	2.2 ± 0.09	10.9 ± 0.5	57.6 ± 1.0
AB30.1	5.5 ± 0.3*	2.7 ± 0.2*	-	7.2 ± 0.4*	0	4.9 ± 0.1*	3.0 ± 0.1**	13.6 ± 0.6*	63.1 ± 0.9*
AB30.3	5.1 ± 0.3*	2.7 ± 0.3*	-	7.0 ± 0.5*	0	4.6 ± 0.2*	3.2 ± 0.08**	14.5 ± 0.3*	63.6 ± 1.2*
AB30.8	4.5 ± 0.4*	3.7 ± 0.2*	-	5.8 ± 0.6**	0	3.4 ± 0.3*	3.1 ± 0.09**	14.2 ± 0.4*	64.9 ± 1.4*
AB30.11	4.4 ± 0.3*	3.4 ± 0.1*	0.4 ± 0.01	6.2 ± 0.7*	0	4.3 ± 0.2*	3.6 ± 0.1**	14.1 ± 0.5*	63.7 ± 0.9*
4 M KOH PC									
WT	1.1 ± 0.05	2.5 ± 0.10	-	65.1 ± 2.1	7.9 ± 0.2	2.4 ± 0.06	6.2 ± 1.2	5.0 ± 0.8	10.1 ± 0.9
V Control-1	1.3 ± 0.06	2.4 ± 0.09	-	64.6 ± 2.3	7.8 ± 0.2	2.5 ± 0.07	6.3 ± 1.0	5.2 ± 0.6	10.3 ± 1.0
AB30.1	1.1 ± 0.04	2.4 ± 0.08	-	47.5 ± 1.9*	5.9 ± 0.2*	2.2 ± 0.06	13.2 ± 1.3*	9.1 ± 0.7*	18.8 ± 1.1**
AB30.3	1.0 ± 0.03	2.2 ± 0.06	-	42.3 ± 1.5*	5.2 ± 0.3*	2.1 ± 0.06	14.6 ± 1.4**	10.7 ± 0.9**	21.8 ± 1.2**
AB30.8	0.9 ± 0.05	2.1 ± 0.07	-	35.7 ± 1.8**	4.3 ± 0.2**	2.0 ± 0.08	17.9 ± 1.7**	12.4 ± 1.0**	24.6 ± 1.3**
AB30.11	1.0 ± 0.05	2.2 ± 0.09	-	37.8 ± 1.6**	4.7 ± 0.3**	2.0 ± 0.09	18.5 ± 0.8**	11.8 ± 0.5**	22.2 ± 0.9**

The amount of sugar is represented as average mol% in each wall extract. AIR (alcohol insoluble residue) was sequentially extracted using 50 mM ammonium oxalate, 50 mM Na_2_CO_3_, 1 M KOH, 4 M KOH, 100 mM sodium chlorite (chlorite) and 4 M KOH PC (post chlorite), hydrolyzed with methanolic HCl, and analyzed by GC. Data are mean ± SE of three biological and two technical replicates. Ital values represent significant difference between WT and *PdGAUT12.1*-KD plants at *P ≤ 0.05, **P ≤ 0.001 significance level (one-way ANOVA followed by Tukey’s multiple comparison test). A dash designates that the amount of sugar was below detection limits.

As expected, extraction of the remaining insoluble wall residues with harsher alkaline solvents such as 1 M and 4 M KOH (conditions which typically enrich for hemicelluloses) released primarily xylose with lesser amounts of glucose, mannose, GlcA, and galactose as well as small amounts of rhamnose, GalA, and arabinonse. The *PdGAUT12.1*-KD 1 M KOH extract contained significantly reduced (35 to 45%) mol% xylose compared to the controls (Table 4). There was also a 35 to 45% reduction in the mol% GlcA and a 60 to 76% reduction in the mol% GalA in these extracts from the *PdGAUT12.1*-KD lines. Similarly, we found a 31 to 41% reduction in xylose in the 4 M KOH extracts from *PdGAUT12.1*-KD RNAi lines. There was also a noticeable increase in mannose, galactose and glucose content in both the 1 M and 4 M KOH extracts. The reduced 35 to 45% mol% xylose and GlcA in the transgenic *Populus* plants (Table 4) agrees with the reduced xylan content in *Arabidopsis irx8/gaut12* mutant plants [8,29]. The even larger reduction (60 to 76%) of GalA in the 1 M KOH extracts of the *PdGAUT12.1*-KD RNAi *Populus* lines suggests that reduced expression of this *GAUT12.1* homolog may also impact a type of pectin that is released from the walls by this extraction.

More tightly bound lignin-associated components in the AIR can be removed by extraction with 100 mM sodium chlorite. There was a 34 to 47% and 24 to 38% reduction of xylose and GalA, respectively, in the chlorite extract from the *PdGAUT12.1*-KD lines compared to the WT controls (Table 4). Similarly, a substantial decrease in arabinose (23 to 39%) and rhamnose (14 to 37%) was observed in this extract from the transgenic lines compared with controls.

After removal of lignin, the residual pellet following the chlorite extractions was treated with a second treatment of 4 M KOH to solubilize additional carbohydrates. Treatment with 4 M KOH after chlorite released primarily xylose. There was a 27 to 45% reduction in the amount of xylose in the post chlorite 4 M KOH extracts from *PdGAUT12.1*-KD lines compared to the controls (Table 4). Similarly, the post chlorite 4 M KOH extract from the *PdGAUT12.1*-KD lines had 27 to 45% reduced amounts of GlcA. No major change in GalA content was observed in this fraction (Table 4).

Taken together, the results indicate that downregulation of *GAUT12.1* in *Populus* wood leads to reductions in a population of pectin and xylan. To compare the mass yield of each sugar from the starting AIR in the *PdGAUT12.1*-KD versus WT cell walls, the data were analyzed as micrograms sugar/milligram AIR. Analyses of the total amount of each sugar in each extract of the starting cell wall (that is, AIR) (Additional file 7) show that only the GalA and rhamnose (Rha) were decreased in each *PdGAUT12.1*-KD cell wall extract compared to WT. These results could be consistent with HG and/or RG-I as the glycan directly affected in the *PdGAUT12.1*-KD lines. However, significant reductions in Ara, Xyl, and GlcA were also observed, with the amount of Ara decreased in five of six extracts and Xyl and GlcA decreased in the four fractions released under the harshest extraction conditions, fractions known to be enriched for hemicelluloses. Thus, the data are also consistent with a role for GAUT12 in xylan synthesis.

#### Glycosyl linkage analyses of fractionated cell walls from *PdGAUT12.1*-KD RNAi plants

Glycosyl residue linkage analyses were carried out to test if the reductions in xylose, GalA, and Rha were associated with xylan, HG, and RG-I, respectively. Specifically, linkage analyses were performed on the ammonium oxalate, sodium carbonate, and 1 M KOH extracts from *PdGAUT12.1*-KD lines AB30.1 and AB30.8 and compared with WT *Populus* (Table 5). The *PdGAUT12.1*-KD plants had 42 to 82% reduced levels of 4-GalA*p* and 55 to 64% reduced amounts of terminal-GalA*p* in the oxalate fractions compared with WT. These same *PdGAUT12.1*-KD fractions, compared to WT, had a 29 to 43% reduction in 2-linked Rha*p*, a linkage found in RG-I. Since the mole percent of 4-linked GalA to 2-linked Rha was approximately 7 (Table 5), taken together, these linkage data indicate that the *PdGAUT12.1*-KD oxalate fraction extracts had reduced amounts of HG and lesser reductions in RG-I. There was also an increase in 4-Xyl*p*, T-Man*p*, 4-Man*p*, 4-Gal*p*, T-Glc*p*, and 4-Glc*p* contents in *PdGAUT12.1*-KD plants compared with WT.

**Table 5:** **Glycosyl linkage analysis of fractionated cell wall fractions from wild type** ***P. deltoides*** **and** ***PdGAUT12.1*** **-KD lines**

**Fractions**	**Ammonium oxalate soluble**			**Na**_**2**_ **CO**_**3**_ **soluble**			**1 M KOH soluble**		
	**WT**	**AB30.1**	**AB30.8**	**WT**	**AB30.1**	**AB30.8**	**WT**	**AB30.1**	**AB30.8**
t-Ara*f*	3.6	1.5	3.3	3.9	1.2	2.6	0.2	0.6	0.9
t-Ara*p*	0.2	0.2	0.3	0.2	0.2	0.2	0.1	0.1	0.1
3-Ara*f*	0.5	0.5	1.0	0.6	0.3	0.5	-	0.2	0.2
3-Ara*p* or 5-Ara*f*	3.2	2.4	2.5	2.2	0.8	1.3	0.2	0.2	0.6
3,4-Ara*p* or 3,5-Ara*f*	1.5	1.3	2.1	-	-	-	-	-	-
t-Rha*p*	0.9	0.6	1.4	1.9	1.0	1.4	0.2	0.4	0.6
2-Rha*p*	2.1	1.2	1.5	3.1	1.6	2.1	0.7	0.5	0.4
3-Rha*p*	-	-	-	-	-	-	1.6	0.9	0.8
2,4-Rha*p*	1.1	1.0	1.4	0.8	0.7	0.8	0.5	1.0	1.0
t-Fuc*p*	0.3	0.2	0.4	0.6	0.4	0.8	0.2	0.5	0.5
t-Xyl*p*	0.4	0.4	0.9	1.6	1.3	1.8	0.9	0.6	0.8
4-Xyl*p*	6.5	9.0	12.5	20.5	21.8	22.1	59.3	38.9	32.3
2,4-Xyl*p*	0.9	1.3	1.5	1.5	1.6	1.6	9.2	5.3	4.1
t-Man*p*	1.2	1.2	2.2	1.8	1.8	2.0	-	-	-
4-Man*p*	17.8	22.9	19.2	19.6	20.1	20.9	6.0	11.0	15.0
4,6-Man*p*	0.7	0.9	0.5	-	-	-	-	-	-
t-GlcA*p*	-	-	2.0	1.6	1.7	2.3	8.5	6.8	3.7
2-GlcA*p*	0.1	0.2	0.2	0.2	0.2	0.2	-	-	-
t-GalA*p*	1.1	0.4	0.5	2.0	1.0	1.4	-	-	-
2-GalA*p*	-	-	0.2	0.9	0.7	1.0	1.8	0.9	0.6
4-GalA*p*	23.8	13.7	4.2	15.5	12.3	8.7	1.5	0.4	0.1
3,4-GalA*p*	-	-	-	0.4	-	0.7	-	-	-
2,4-GalA*p*	0.1	0.1	0.1	0.1	-	-	-	-	0.1
t-Gal*p*	2.9	2.1	2.7	3.2	3.1	3.0	1.2	2.9	3.1
2-Gal*p*	0.4	0.2	0.3	0.6	-	-	-	0.4	0.6
4-Gal*p*	-	-	3.3	-	-	2.8	-	-	0.1
6-Gal*p*	0.4	1.1	0.8	-	-	0.8	0.5	0.8	1.0
3,4-Gal*p*	-	1.2	1.0	0.3	-	-	-	-	-
2,4-Gal*p*	0.2	0.3	0.3	0.2	-	-	-	0.1	0.2
t-Glc*p*	3.3	3.4	4.3	3.6	2.9	3.2	-	2.6	2.8
2-Glc*p*	0.3	0.3	0.5	0.1	0.1	0.2	-	-	-
4-Glc*p*	26.2	31.6	28.3	12.9	25.5	17.7	7.4	24.4	29.8
4,6-Glc*p*	0.4	0.7	0.6	-	-	-	-	0.4	0.7

All numbers are mole percentages. A dash designates that the amount of sugar was below detection limits. Glycosyl linkage analyses were provided by the CCRC Analytical Services.

In the carbonate fractions, we observed 21 to 44% reduced amounts of 4-GalA*p* and 30 to 50% reduced levels of terminal-GalA*p* in the *PdGAUT12.1*-KD lines compared to WT (Table 5). The 2-linked Rha*p* content was also reduced by 32 to 48% in the carbonate extracts of *PdGAUT12.1*-KD lines compared to WT, although again, the 2-linked Rha was only 8 mole% of the GalA content. There was a corresponding increase in the amount of 4-Xyl*p* and 4-Glc*p* in the *PdGAUT12.1*-KD carbonate fraction. These data are consistent with the hypothesis that the *Populus GAUT12.1* gene encodes a glycosyltransferase that makes a subfraction of HG.

A significant reduction of 34 to 46% in 4-Xyl*p* content in the 1 M KOH extract of the *PdGAUT12.1*-KD line corroborates the results from the glycosyl residue composition analysis (Table 5) and indicates that the 1 M KOH fraction of the *PdGAUT12.1*-KD plants had reduced amounts of xylan (4-Xyl*p*). A 73 to 93% reduction in 4-GalA*p* content in the KD lines indicates a reduction in the amount of HG compared to WT. Also, a 50 to 67% reduction of 2-GalA*p* and a 44 to 50% reduction in 3-Rha*p* suggests a simultaneous reduction in the xylan reducing end sequence [39]. The reduced content of 4-Xyl*p* in this 1 M KOH extract along with reduced amounts of 2-GalA*p* and 3-Rha*p* would be consistent with a role for GAUT12 in the addition of GalA into xylan reducing end oligosaccharide sequence in *Populus*. The greater mole percent reduction in 4-GalA*p* compared to 2-GalA*p* would be consistent with a role for GAUT12 in synthesizing a GalA-containing moiety, possibly HG, required for the formation of xylan containing the reducing end oligosaccharide or linking the xylan to another structure in the wall. These overall results show that GAUT12 knockdown leads to defects in pectin and xylan production in *Populus*.

#### Glycome profiling of fractionated cell walls from *PdGAUT12.1*-KD plants

**Figure 9:**
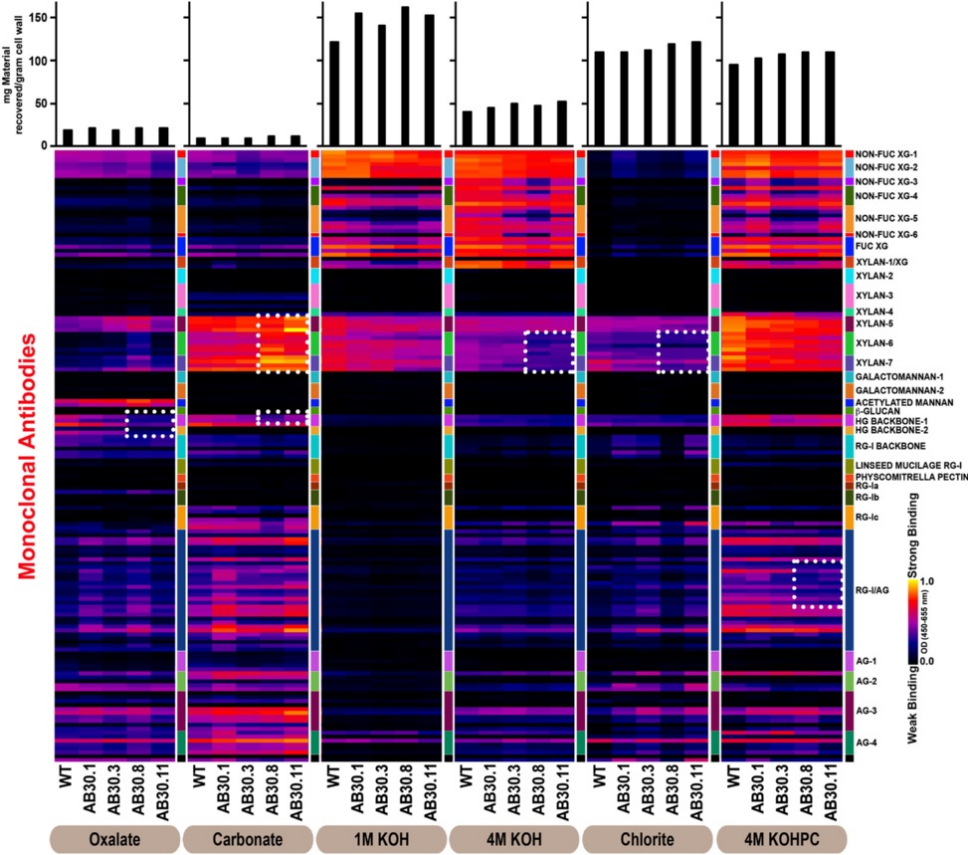
**Glycome profiling of** ***PdGAUT12.1*** **-KD** ***Populus*** **plants.** Sequential extracts of cell walls (AIR) were prepared from WT and *PdGAUT12.1*-KD *Populus* lines **(**AB30.1, AB30.3, AB30.8, and AB30.11) using increasingly harsh reagents as explained in methods. The extracts were screened by ELISA using 155 mAbs directed against epitopes present on most major non-cellulosic plant cell wall glycans (Additional file 8). The resulting binding response data are represented as heatmaps using a white-red-dark-blue scale indicating the strength of the ELISA signal (white, red, and dark-blue colors depict strong, medium, and no binding, respectively). The mAbs are grouped based on the cell wall glycans they predominantly recognize as depicted in the panel at right hand side of the figure. The gravimetric amounts of materials extracted from the wall by each extraction reagent are depicted as bar graphs at the top of the heatmaps. The dotted boxes show major regions of the profiles where reduced antibody binding was observed in extracts of *PdGAUT12.1*-KD walls.

In an effort to better understand the specific defects in xylan and pectin, glycome profiling of sequential extracts from WT and *PdGAUT12.1*-KD walls was performed. Glycome profiling is an ELISA-based technique in which monoclonal antibodies directed against distinct cell wall carbohydrate epitopes are used to identify differences in specific carbohydrate structures present in sequential cell wall extracts [54-56]. Glycome profiling using a panel of 155 monoclonal antibodies directed against diverse cell wall matrix polysaccharide epitopes (Additional file 8) revealed changes in the extractability of three types of wall polysaccharides (Figure 9). In general, a marginal increase in the amounts of 1 M KOH extractable material (as noted in the bar graphs above the heat maps) was observed for all *PdGAUT12.1*-KD lines, suggesting an enhanced extractability of some hemicelluloses in these lines compared to the WT. A marginally enhanced abundance of xylan epitopes (recognized by the xylan-6 and 7 mAb groups) was observed in carbonate extracts from two GAUT12-KD lines (AB30.8 and AB30.11), suggesting that a portion of cell wall xylan is more easily extracted in the GAUT12-KD lines. Concomitantly, these two lines exhibited reduced abundance of these xylan epitopes in the 4 M KOH and chlorite extracts and a lesser reduction in the 1 M KOH extract. These results suggest that a portion of xylan in the *PdGAUT12.1*-KD lines (AB30.8 and AB30.11) was held less tightly in the walls and was recovered in the carbonate fraction. In these *PdGAUT12.1*-KD lines, there was also a reduction in HG epitopes in the oxalate extract, carbonate, and 4 M KOH postchlorite fractions, as well as a slight reduction of some RG-I/AG mAb binding in the 4 M KOH postchlorite extract.

The combination of results obtained from glycosyl residue and linkage composition analyses of total AIR together with sugar composition analyses, glycome profiling, and total yield of sequential wall extracts indicate the following. (1) The only sugars consistently and statistically reduced in total AIR from *PdGAUT12.1*-KD lines were xylose and GalA, suggesting that GAUT12 may function in the addition of one or both of these into one or more wall polymers. (2) The only sugars consistently and statistically reduced in all the wall fractions, both the pectin- and xylan-enriched fractions, were GalA and rhamnose. Combined with linkage data, these results suggest that a pectin-like HG or HG/RG-I structure is either synthesized by GAUT12, or is part of the polymer synthesized by GAUT12. The significant reduction in xylose content in the four hemicellulose-enriched wall extracts suggests that xylan or a xylan-containing moiety may also be either the, or a part of, the polymer synthesized by GAUT12. (3) *GAUT12.1*-KD walls are more easily extracted as evidenced by the reduced yield of insoluble AIR pellet and the recovery of increased amounts of material in the sequential cell wall extracts. These results strongly support the hypothesis that the structure(s) synthesized by GAUT12 are part of the basic wall infrastructure. (4) Glycome profiling confirms that both xylan and HG along with some rhamnogalacturonan/arabinogalactan epitopes are affected in the *PdGAUT12.1*-KD lines, with losses in the HG and RG/AG epitopes and an increased ease of extraction of the xylan epitope.

## Conclusions

We applied a genetic engineering approach to modify *GAUT12.1* expression in *Populus*. Eleven transgenic lines were obtained that had reduced *PdGAUT12.1* transcript expression and an accompanying 25 to 47% reduction in GalA, 17 to 30% reduction in xylan content, and 3 to 7% increase in sugar release across the lines. We also found a significant increase in plant height (12 to 52%) and stem diameter (12 to 44%) and leaf size (52 to 117%).

It has been reported that removal of hemicellulose, especially xylose, xylo-oligomers, and xylan from lignocellulosic materials prior to enzymatic hydrolysis increases enzyme accessibility, conversion rates, and hydrolysis yield of plant biomass [41,42,57]. Similarly, pectin degradation has been shown to enhance saccharification [43]. Thus, a possible explanation for the greater sugar release in the *P. deltoides PdGAUT12.1*-KD lines is that the reduction of 17 to 30% xylose and 25 to 47% GalA associated with xylan and HG in the cell wall leads to an increased accessibility of the biomass to enzymes and results in greater conversion and sugar release. These data support our hypothesis that downregulation of *GAUT12.1* reduces recalcitrance in *Populus.*

There was also a significant increase in phloem tissue, xylem fiber cell size, and xylem vessel diameter size in *PdGAUT12.1*-KD wood. The larger leaf area in both developing and expanded leaves, the larger individual palisade and spongy parenchyma cells, the higher RWC, and the increased phloem and wider vessels in the *PdGAUT12.1*-KD lines may explain the greater growth and biomass yield in *PdGAUT12.1*-KD RNAi *Populus* lines.

We also demonstrated that *PdGAUT12.1*, which is highly expressed in developing xylem (Figure 2B), can complement the phenotype of Arabidopsis *irx8* mutants (Figure 8), indicating that it is a functional homolog of the Arabidopsis *GAUT12. PdGAUT12.2*, which is expressed more broadly and at lower levels (Figure 2C), showed only partial complementation in the same experiments, suggesting that it might have functionally diverged from *PdGAUT12.1*.

The substantial decrease in GalA and xylose content in the *PdGAUT12.1*-KD cell walls and the content of fractionated walls as determined by glycosyl residue composition, linkage analyses, and glycome profiling support the hypothesis that reduced *GAUT12.1* transcript levels affect both pectin and xylan biosynthesis in *Populus.* Sequential extraction of cell walls with increasingly harsh solvents in an effort to identify the specific polymers affected revealed an 11 to 17% increase in extractable material in walls of the *PdGAUT12.1*-KD lines compared to the controls and a concomitant 8 to 13% reduced mass of the final insoluble pellet, indicating a loosened wall structure in the *PdGAUT12.1*-KD lines that enables more facile extraction. Analyses of sequential cell wall extracts confirmed a reduction of both GX and the pectins HG and RG-I in extracts from *PdGAUT12.1*-KD RNAi lines. These transgenic lines had a higher S/G ratio but no major change in the total lignin content. Since no major change in the total lignin was observed, the data support the hypothesis that GAUT12.1 synthesizes a wall structure that is either required for deposition of xylan and pectin or that contains xylan and pectin and that when this structure is not made, the wall is held together less tightly, leading to increased sugar release and increased growth. These results indicate that a systematic biotechnological manipulation of pectin and xylan biosynthetic genes in woody *Populus* can be used to develop less recalcitrant woody biomass with increased growth potential that can be more easily deconstructed and fermented into biofuels. The exact structure synthesized by GAUT12.1 remains to be determined, but the data presented here suggest it is a core architectural structure in *Populus* wood cell walls.

## Methods

### Plant materials and growth conditions

*P. deltoides* plants were grown in Fafards 3B Soil mix (GroSouth Inc, Atlanta, GA, USA) with osmocote (250 ml/bag 79 L 3B soil), bone meal (84 ml/bag 79 L 3B soil), gypsum (84 ml/bag 79 L 3B soil), and dolomite/limestone (42 ml/1bag 3B soil) in the greenhouse under a 16-h-light/8-h-dark cycle at 25 to 32°C, depending on the season. All WT, vector control, and transgenic plants were grown for 9 months and fertilized weekly with Peters 20-10-20 (nitrogen-phosphorus-potassium; GroSouth Inc, Atlanta, GA, USA). The bottom 6 cm of the stem from WT, vector control, and transgenic 9-month-old plants was harvested and the bark removed by peeling with a razor. The peeled stem samples were air-dried and the pith was removed using a hand drill. The remaining stem material was milled to a particle size of 20 mesh (0.85 mm) using a Wiley Mini-Mill (model number 3383 L10, Thomas Scientific). Ground material was used for analytical pyrolysis, saccharification, and cell wall composition analyses.

Arabidopsis plants were grown on soil in a controlled-environment chamber (Conviron, Pembina, ND, USA) under a 14-h-light/10-h-dark cycle at 19°C during the light and 15°C during the dark. The light intensity was 150 μEm^−2^/s and relative humidity was maintained at 50%. T-DNA insertions were confirmed using primers from genomic regions flanking the T-DNA and the general T-DNA left border primer (Additional file 5). Arabidopsis *Col-0* and heterozygous *irx8-5* (SALK_044387) plants were transformed via the floral dip method [58], and transgenic plants harboring BASTA-resistant plasmids were selected on MS media plates containing 10 mg/l DL-phosphinothricin (PhytoTechnology Laboratories G523) and 100 mg/l timentin (PhytoTechnology Laboratories T869). Timentin inhibits residual Agrobacterium growth resulting from the dipping transformation. T1 transgenic plants harboring the construct in *Col-0* and *irx8-5* homozygote mutant backgrounds were selected by genotyping PCR (Additional file 5) and used for complementation analyses.

### Vector construction and transformation of *Populus*

The *PtGAUT12.1* RNAi construct used for transformation of *P. deltoides* was generated by cloning a 168-bp fragment from the 3′-untranslated region of *POPTR_0001s44250/Potri.001G416800*, which was amplified via PCR from a *P. trichocarpa* cDNA library using the forward primer 5′-CACCCCCGGGGAGAGTTCTTCACAAATCCA-3′ and the reverse primer 5′- TCTAGAAAATCCATAACATGCTTAATTTCTC-3′. The integrity of the fragment was verified by DNA sequencing (ACGT, Inc., Wheeling, IL, USA) after cloning into the Gateway® entry vector, pENTR/D-TOPO (Life Technologies, Grand Island, NY, USA). The fragment was transferred to a binary Gateway® destination plasmid, pAGSM552 (GenBank accession KP259613) using LR Clonase II recombination (Life Technologies). The resulting binary transformation vector, pAGW641 (GenBank accession KP259612), was transformed into *A. tumefaciens* strain EHA105 via electroporation. The RNAi cassette comprised the Arabidopsis UBIQUITIN3 promoter, inverted repeats of the *PtGAUT12.1* fragment flanking a spacer, and the nopaline synthase terminator. The spacer was a 1.3-kb fragment of the *Petunia hybrida chsA* intron. *P. deltoides* genotype WV94 was transformed using a modified Agrobacterium-based method [59,60]. Shoots regenerated from isolated calli were tested using PCR to verify the presence of the *GAUT12.1* construct.

### Generation of constructs for complementation of Arabidopsis *irx8* mutant

Arabidopsis *GAUT12* coding sequence (CDS) was amplified from Arabidopsis cDNA using primers whose sequences are provided in Additional file 5. Poplar GAUT12 cDNAs were generated by reverse transcription-PCR as follows: approximately 1 μg of RNA harvested from WT *P. deltoides* stems was reverse transcribed into first-strand cDNA with oligo-dT primers. This library was diluted tenfold and used as a template for amplifying the predicted coding sequences of *PdGAUT12.1*(*POPTR_0001s44250*, Phytozome 8.0*/Potri.001G416800*, Phytozome 10.0) and *PdGAUT12.2* (*POPTR_0011s13600*, Phytozome 8.0*/Potri.011G132600*, Phytozome 10.0) by a nested PCR with specific primers designed on the basis of the *P. trichocarpa* genome sequence available at Phytozome (http://www.phytozome.net/, http://phytozome.jgi.doe.gov/). PCR products were gel-purified and ligated into pGEM®-T Easy vector (Promega, Madison, WI, USA) and confirmed by sequencing. BamHI and ApaI restriction sites included in the second-round primers were used for lacing the coding sequences downstream of the 35S promoter of a T-DNA derived from pCAMBIA3300. T-DNAs were moved into *Agrobacterium tumefaciens* strain GV3101 by electroporation and the cultures used for plant transformation as described above.

### RNA isolation and quantitative real-time PCR

Plant tissues used to isolate RNA for transcript analysis were *P. deltoides* young leaves, mature leaves, stems of 6 to 8 internodes, petioles pooled from internodes 1 and 2, cambium samples lightly scraped from frozen peeled bark, and xylem samples scraped from debarked frozen stem. All of the tissues were ground to a fine powder in liquid nitrogen. Total RNA was isolated from 100 to 150 mg frozen powder from three individual tissue samples using hexadecyl-trimethylammonium bromide (CTAB; 2% CTAB, 2% polyvinylpyrolidone, 100 mM Tris-HCl, 25 mM EDTA, 2 M NaCl, 0.5 g L21 spermidine, and 2% β-mercaptoethanol, pH 8.0) [61] and purified using columns from an RNeasy Plant Mini Kit (Qiagen, Venlo, Netherlands; 74903). Genomic DNA was removed by treatment with DNase (Ambion) and first-strand cDNA was synthesized from 1 μg total RNA using SuperScript III First-Strand Synthesis Super Mix (Invitrogen). Primers for real-time PCR were designed using Beacon Designer (v 2.1, Premier Biosoft International, Palo Alto, CA, USA) and tested for efficiency and specificity. The least conserved region of the *PtGAUT12.1 and PtGAUT12.2* genes in the poplar genome (Phytozome 8.0/Phytozome 10.0) was used for primer design. The primers used for this analysis were as follows: *GAUT12.1* F 5′-GGTCGAGCAAAGCCTTGGCTAGATATAGC-3′ and *GAUT12.1* R 5′-AGATGGCCTAATATGACAGCCCTTTA-3′ yielding a PCR product of 111 bp and *GAUT12.2* F 5′-CATTTCAATGGTCGAGCAAAGCCTTGGC-3′ and *GAUT12.2* R 5′-GACAGCCCGTAATGAACTTGTCAGA-3′ yielding a PCR product of 106 bp. The *18S rRNA* reference gene was used to amplify a product of 74 bp (*18S* F 5′-AATTGTTGGTCTTCAACGAGGAA-3′ and *18S* R 5′-AAAGGGCAGGGACGTAGTCAA-3′). The PCR reactions were performed in triplicate in a 96-well plate format using iQ TM SYBR Green Supermix (Bio-Rad, Hercules, CA, USA; 170-8882) in a CFX96 TM Real-Time PCR Detection System (Bio-Rad) according to the manufacturer’s instructions. The relative transcript levels were calculated by normalizing target gene expression to control gene expression, where expression of the control gene was set to 1 [62,63].

### Plant growth analysis, water status, and measurement of dry weight

After the WT, vector control and transgenic plants developed proper roots in BTM media, all plants were transferred to soil in the greenhouse. Twelve to 15 plants per line were measured for plant height and radial stem growth. All plants were grown for nine months and initial plant stem growth was measured at three months. To determine the sizes of leaves from controls and transgenic lines at different developmental stages, the length and width of every fifth successive leaf from the apex was measured from three-month-old plants. Every 5^th^, 6^th^ and 7^th^ leaf from the apex of six WT and six transgenic plants was collected and measured for leaf area to compare developing leaf growth. Similarly, the 20^th^ to 40^th^ leaves from the apex of five transgenic lines were collected and measured to compare fully expanded leaf areas.

Plant water status was measured as RWC of WT and *PdGAUT12.1*-KD plants [64]. The RWC is expressed as RWC (%) = [(FM − DM)/(TM − DM)] × 100, where, FM, DM, and TM are the fresh, dry, and turgid mass, respectively. Leaves were detached from the plants and used to measure FW. To obtain the turgid mass (TM), the detached leaves were rehydrated at 4°C for 24 h. The rehydrated leaves were placed in a pre-heated oven at 70°C for 72 h to measure the dry mass (DM). For the dry matter measurements, two sets of three individual three month old plants (above ground plant parts, that is, entire shoots) were harvested and each set was dried separately at 70°C for 5 days, weighed and mass values averaged (*N* = 6).

### Preparation of AIR cell wall and cell wall fractionations

The ground biomass was sequentially extracted with 80% (*v*/*v*) ethanol, 100% ethanol, and chloroform/methanol (1:1[*v*/*v*]), and after centrifugation, the resulting cell wall residue (AIR) was vacuum-dried for 24 h at room temperature. Sequential fractionations were carried out at 10 mg/ml based on the starting dry weight of AIR in order to isolate fractions enriched for different types of cell wall components. AIR wall was suspended at 25°C in 50 mM ammonium oxalate, pH 5.0 for 24 h with constant shaking at 100 rpm. After incubation, the mixture was centrifuged at 4,000 *g* for 15 min at room temperature and the supernatant saved as the ammonium oxalate extract. The residual pellet was subsequently washed three times with the same volume of deionized water, centrifuged, and the supernatant discarded. The pellet was treated at 25°C with 50 mM Na_2_CO_3_, pH 10.0 (containing 0.5% (*w*/*v*) sodium borohydride) with constant shaking (100 rpm). After centrifugation, the supernatant was saved as the sodium carbonate extract. The washed residual pellet was re-suspended in 1 M KOH with 1% (*w*/*v*) sodium borohydride and incubated at 25°C for 24 h with constant shaking at 100 rpm. After incubation, the suspension was centrifuged at 4,000 *g* for 15 min, the supernatant collected and labeled as 1 M KOH extract, and stored at 4°C. The residual pellet from the 1 M KOH fraction was re-suspended in 4 M KOH with 1% (*w*/*v*) sodium borohydride and incubated at 25°C for 24 h with constant shaking at 100 rpm. After incubation, the suspension was centrifuged at 4,000 *g* for 15 min and the supernatant collected, labeled as 4 M KOH extract and stored at 4°C. The residual pellet from the 4 M KOH fraction was treated with 100 mM sodium chlorite at 70°C for 3 h to break down lignin polymers. After centrifugation, the supernatant was collected and labeled as chlorite extract. The residual pellet left after the sodium chlorite treatment was treated with 4 M KOH containing 1% (*w*/*v*) sodium borohydride extraction at 25°C for 24 h with constant shaking (100 rpm) and the supernatant collected and labeled as 4MKOH PC. The 1MKOH, 4MKOH, and 4MKOH PC fractions were neutralized using glacial acetic acid, dialyzed against six changes of de-ionized water at room temperature for 72 h, and lyophilized [55].

### Glycosyl residue composition of AIR and fractionation of *Populus* stem cell walls

Glycosyl residue composition analysis of stems from 9-month-old WT, vector control, and *PdGAUT12.1*-KD plants was carried out by preparation and analysis of trimethylsilyl (TMS) derivatives of the monosaccharide methyl glycosides produced from the samples by acidic methanolysis [65,66]. The stem AIR (approximately 2 mg) and fractionated cell walls (100 to 300 μg) were aliquoted into individual tubes, and 20 μg of inositol was added as an internal standard. The samples were lyophilized and dry samples hydrolyzed for 18 h at 80°C in 1 M methanolic-HCl. The samples were cooled, evaporated under a stream of dry air, and further dried two additional times with anhydrous methanol. The walls were derivatized with 200 μl of TriSil Reagent (Pierce-Endogen, Rockford, IL, USA) and heated to 80°C for 20 min. The cooled samples were evaporated under a stream of dry air, resuspended in 3 ml hexane, and filtered through packed glass wool. The dried samples were resuspended in 150 μl hexane and 1 μl of sample was injected into the gas chromatograph-mass spectrometer (GC-MS). GC/MS analysis of the TMS methyl glycosides was performed on an Agilent 7890A GC interfaced to a 5975C MSD using a Supelco EC-1 fused silica capillary column (30 m × 0.25 mm ID) and helium as carrier gas.

### Glycosyl residue linkage analysis

For glycosyl residue linkage analysis, the samples were permethylated, reduced, re-permethylated, depolymerized, reduced, and acetylated. The resulting partially methylated alditol acetates (PMAAs) were analyzed by gas chromatography-mass spectrometry (GC-MS) as previously described [66].

The fractionated wall samples from WT and *PdGAUT12.1*-KD lines were first suspended in 200 ul dimethyl sulfoxide and the samples stirred for 2 days at 25°C. The samples were permethylated using potassium dimsyl anion and iodomethane. The permethylated uronic acids were reduced using lithium borodeuteride and then permethylated again by the method described earlier [67] with sodium hydroxide and methyl iodide in dry DMSO. This additional permethylation was to insure complete methylation of the polymer. Following sample workup, the permethylated material was hydrolyzed using 2 M trifluoroacetic acid (2 h in sealed tube at 121°C), reduced with NaBD_4_, and acetylated using acetic anhydride/TFA. The resulting PMAAs were analyzed on an Agilent 7890A GC interfaced to a 5975C MSD (mass selective detector, electron impact ionization mode). Separation was performed on a Supelco SP-2380 fused silica capillary column (30 m × 0.25 mm ID).

### Analytical pyrolysis for lignin analysis

All *Populus* biomass samples were analyzed by analytical pyrolysis for lignin content and S/G ratio. A commercially available molecular beam mass spectrometer (MBMS) designed specifically for biomass analysis was used for pyrolysis vapor analysis [68-70]. Approximately 4 mg of air-dried 20-mesh biomass was introduced into the quartz pyrolysis reactor via 80 μl deactivated stainless steel Eco-Cups provided with the autosampler. Mass spectral data from *m*/*z* 30 to 450 were acquired on a Merlin Automation data system version 3.0 using 17 eV electron impact ionization. S/G ratios were determined by summing the syringyl peaks 154, 167, 168, 182, 194, 208, and 210 and dividing by the sum of guaiacyl peaks 124, 137, 138, 150, 164, and 178 (Additional file 9). Several lignin peaks were omitted in the syringyl or guaiacyl summations due to individual peaks having associations with both S and G precursors [68]. Lignin estimates were determined by summing the intensities of the major lignin precursors listed in Additional file 9.

### Saccharification assay

High-throughput thermochemical pretreatment and enzymatic hydrolysis of biomass was carried out as previously described [71,72]. Briefly, ground *Populus* biomass was extracted with glucoamylase (Spirizyme Ultra - 0.25%) and alpha-amylase (Liquozyme SC DS - 1.5%) in 0.1 M sodium acetate (24 h, 55°C, pH 5.0) to remove possible starch content (16 ml enzyme solution per 1 g biomass) [73]. This was followed by an ethanol Soxhlet extraction for an additional 24 h to remove extractives. After drying overnight, 5 mg (±0.5 mg) was weighed in triplicate into individual wells of custom-made 96-well format Hastelloy reactor plates. Water was added (250 μl), the samples sealed with silicone adhesive-backed Teflon tape, clamped tightly, and treated at 180°C for 17.5 min in a customized steam reactor. Once cooled, 40 μl of buffer-enzyme stock was added. The buffer-enzyme stock was 8% CTec2 (Novozymes) in 1.0 M sodium citrate buffer. The samples were gently mixed and left to statically incubate at 50°C for 70 h. After 70 h of incubation, an aliquot of the saccharified hydrolysate was diluted and analyzed using Megazyme’s GOPOD (glucose oxidase/peroxidase) and XDH assays (xylose dehydrogenase), using mixed glucose/xylose standards.

### Tissue fixation, embedding and microscopy

Both leaf (6th leaf from the apex) and stem (10th and 20th internode from the apex) of *P. deltoides* tissues were cut into small pieces (~3 mm) with a razor blade and immediately fixed in 25 mM sodium phosphate buffer pH (7.1) with 4% (*v*/*v*) paraformaldehyde, 0.5% (*v*/*v*) glutaraldehyde, and 0.02% (*v*/*v*) Triton X-100 for 24 h at 4°C. Tissues were washed three times for 30 min each with 25 mM sodium phosphate buffer, pH 7.1, followed by three washes with water. In the next step, the samples underwent a graded ethanol series (35%, 50%, 70%, 95%, and 100% [*v*/*v*]) wash for 25 min at each step (one time) to dehydrate the tissue. The 100% ethanol step was repeated two times to remove as much water as possible from the tissue. The samples were infiltrated with LR White embedding resin (Ted Pella) as follows: 1:3 (LR White: 100% ethanol) for 24 h (one time); 1:1 (LR White: 100% ethanol) for 24 h (one time); 3:1 (LR White: 100% ethanol) for 24 h (one time); and LR White (100%, no ethanol) for 24 h (three times). After the last resin change, the samples were transferred into gelatin capsules with fresh LR White. The gelatin capsules filled with resin were polymerized under 365-nm UV light for 48 h at 4°C. Sections, approximately 1 um in thickness, were cut with a diamond Histo-knife on a Reichert-Jung Ultracut S ultramicrotome (Leica Microsystems, Wetzlar, Germany) and mounted onto pre-coated slides (Colorfrost/Plus, Fisher Scientific, Waltham, MA, USA). The mounted samples were stained with 1% toluidine blue for light microscopy. Images were captured using a Nikon DS-Ri1 camera (Nikon, Melville, NY, USA) and NIS-Elements Basic Research software.

### Maceration of xylem

The bottom part of the stem (approximately 6 cm) from 9-month-old WT, vector control, and *PdGAUT12.1*-KD lines was harvested and debarked. Stem segments approximately 10 mm in length and 3 mm in diameter were used for maceration. The debarked stem segments were macerated with little modification in 50% glacial acetate acid and 3% hydrogen peroxide at 100°C for 12 h [74]. The samples were washed three times in water and the pH neutralized. The samples were suspended in 70% ethanol and vigorously shaken to release individual xylem fibers and vessel cells. The length and width of approximately 80 fibers and 60 vessel elements were measured from images captured using a Nikon DS-Ri1 camera (Nikon, Melville, NY) and NIS-Elements Basic Research software.

### Glycome profiling

Glycome profiling of the sequential extracts prepared above was carried out as described previously [55]. Plant glycan-directed monoclonal antibodies [54-56] were from laboratory stocks (CCRC, JIM, and MAC series) at the Complex Carbohydrate Research Center (available through CarboSource Services; http://www.carbosource.net) or were obtained from BioSupplies (Australia) (BG1, LAMP). A description of the mAbs used in this study can be found in Additional file 8, which includes links to a web database, Wall*MAb*DB (http://www.wallmabdb.net) that provides detailed information about each antibody.

### Statistical analysis

Statistical analysis was performed using Statistica 5.0. The significance of differences between control and transgenic was analyzed using a one-way ANOVA followed by Tukey’s multiple comparison test or *post hoc* Bonferroni corrected *t*-test.

## Additional files

Additional file 1:
**Relationship of GAUT12.1 transcript expression to plant height and diameter.**
Additional file 2:
**Physiological measurements on** ***PdGAUT12.1*** **-KD lines.**
Additional file 3:
**Downregulation of** ***GAUT12.1*** **leads to increased numbers of palisade parenchyma cells.**
Additional file 4:
**Leaf anatomy of** ***PdGAUT12.1*** **-KD plants.**
Additional file 5:
**List of primers used for complementation study.**
Additional file 6:
**Mass of extracts recovered upon extraction of** ***PdGAUT12.1*** **-KD AIR.**
Additional file 7:
**Monosaccharide composition and total carbohydrate of cell wall fractions from** ***PdGAUT12.1*** **-KD and control plants.**
Additional file 8:
**List of cell wall** **glycan-directed** **monoclonal antibodies (mAbs) used for glycome profiling analyses.**
Additional file 9:
**Lignin peak and precursor assignments of analytical pyrolysis mass spectra.**

## Abbreviations

AIR: Alcohol-insoluble residue
GalA: Galacturonic acid
GAUT1: Galacturonosyltransferase1
GAUT12: Galacturonosyltransferase12
GlcA: Glucuronic acid
GT8: Glycosyltransferase 8
GX: Glucuronoxylan
HG: Homogalacturonan
MBMS: Molecular beam mass spectrometer
MeGlcA: Methyl glucuronic acid
RG: Rhamnogalacturonan
TMS: Trimethylsilyl
WT: Wild type
