# Supplementary figures and images for: Downregulation of GAUT12 in Populus deltoides by RNA silencing results in reduced recalcitrance, increased growth and reduced xylan and pectin in a woody biofuel feedstock
Source: Biotechnol Biofuels. 2015 Mar 12;8:41. doi: 10.1186/s13068-015-0218-y (PMC4369864; doi:10.1186/s13068-015-0218-y)

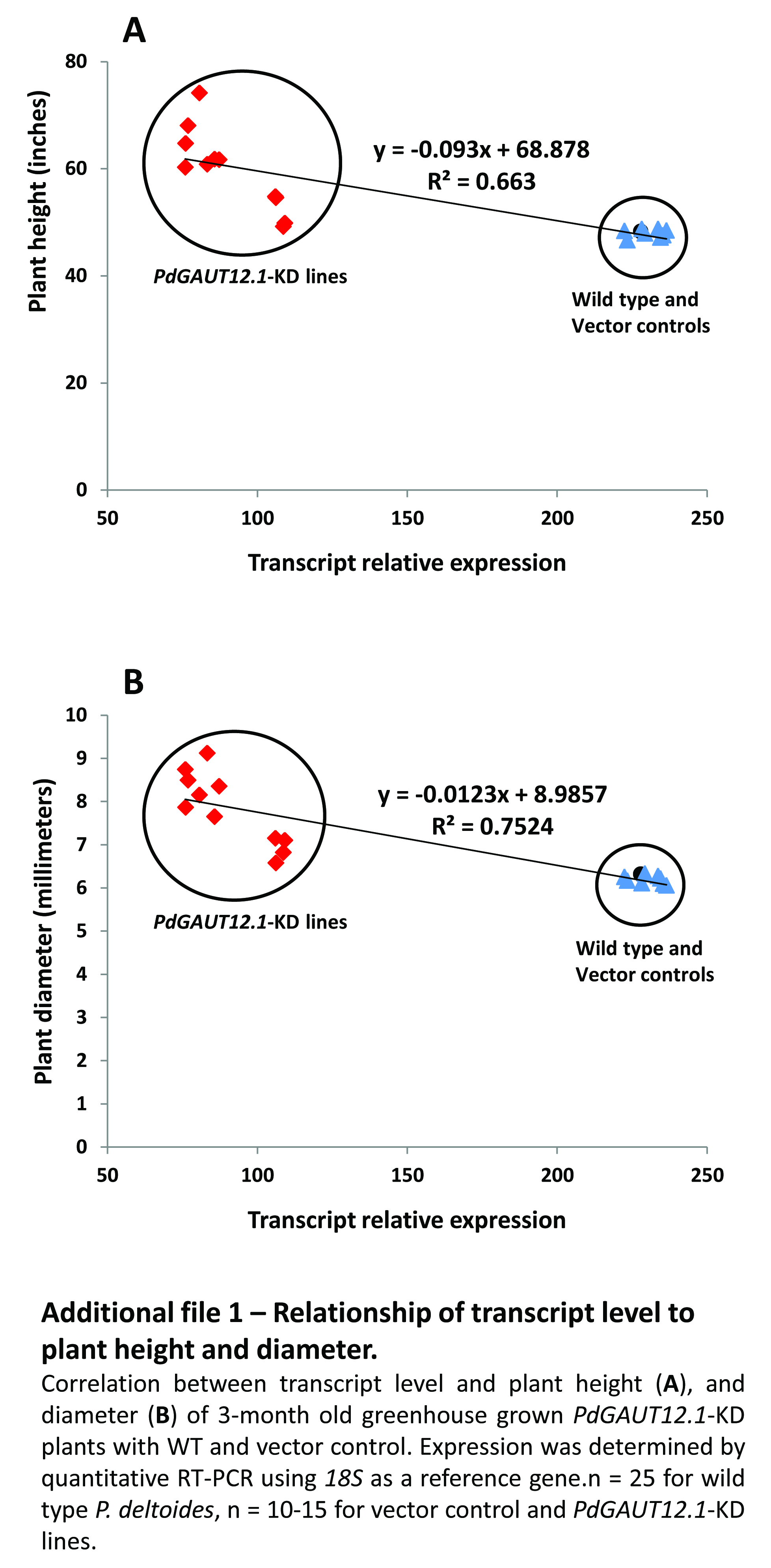

Supplement: Additional file 1: — Relationship of GAUT12.1 transcript expression to plant height and diameter. [file 13068_2015_218_MOESM1_ESM.tiff]

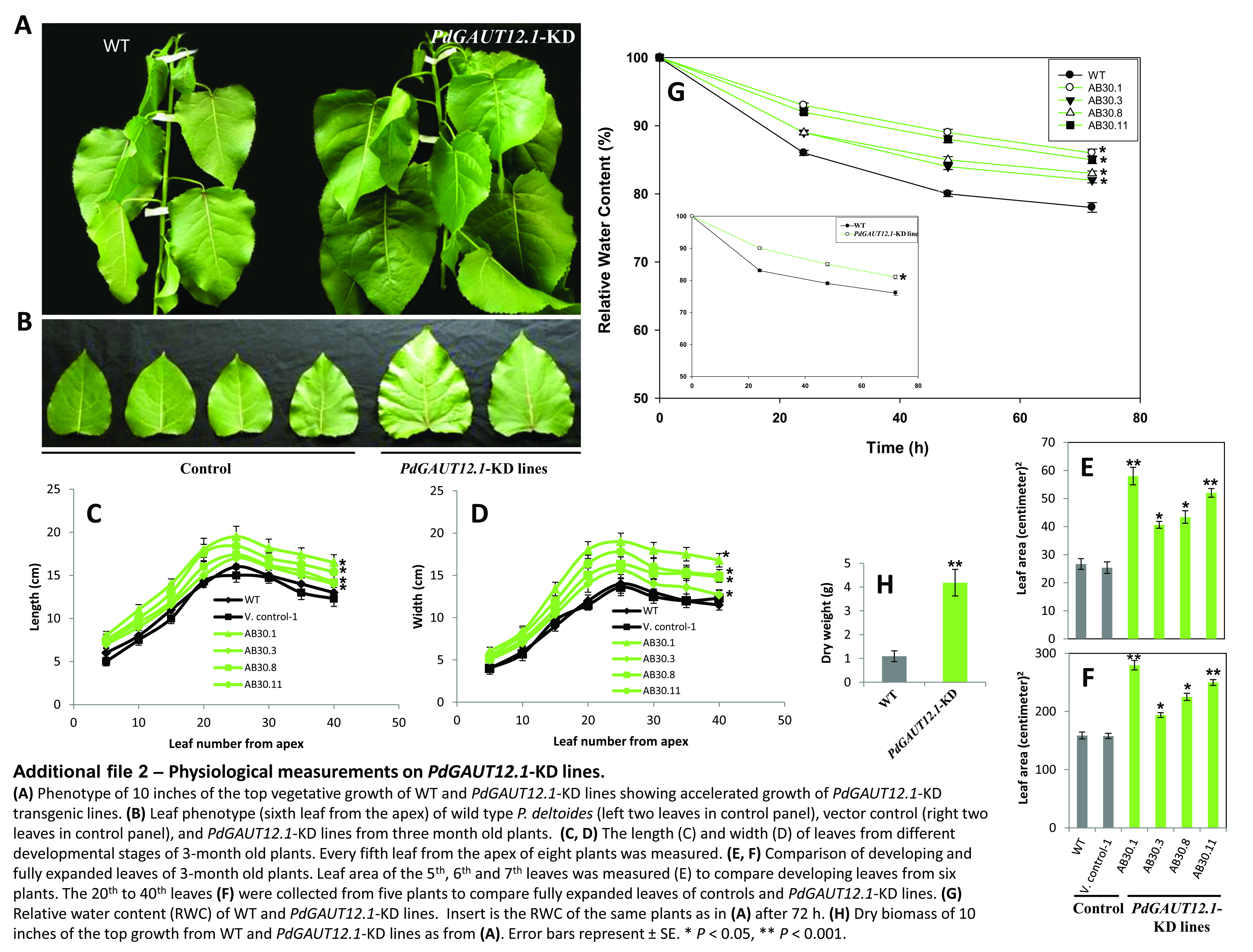

Supplement: Additional file 2: — Physiological measurements on PdGAUT12.1 -KD lines. [file 13068_2015_218_MOESM2_ESM.tiff]

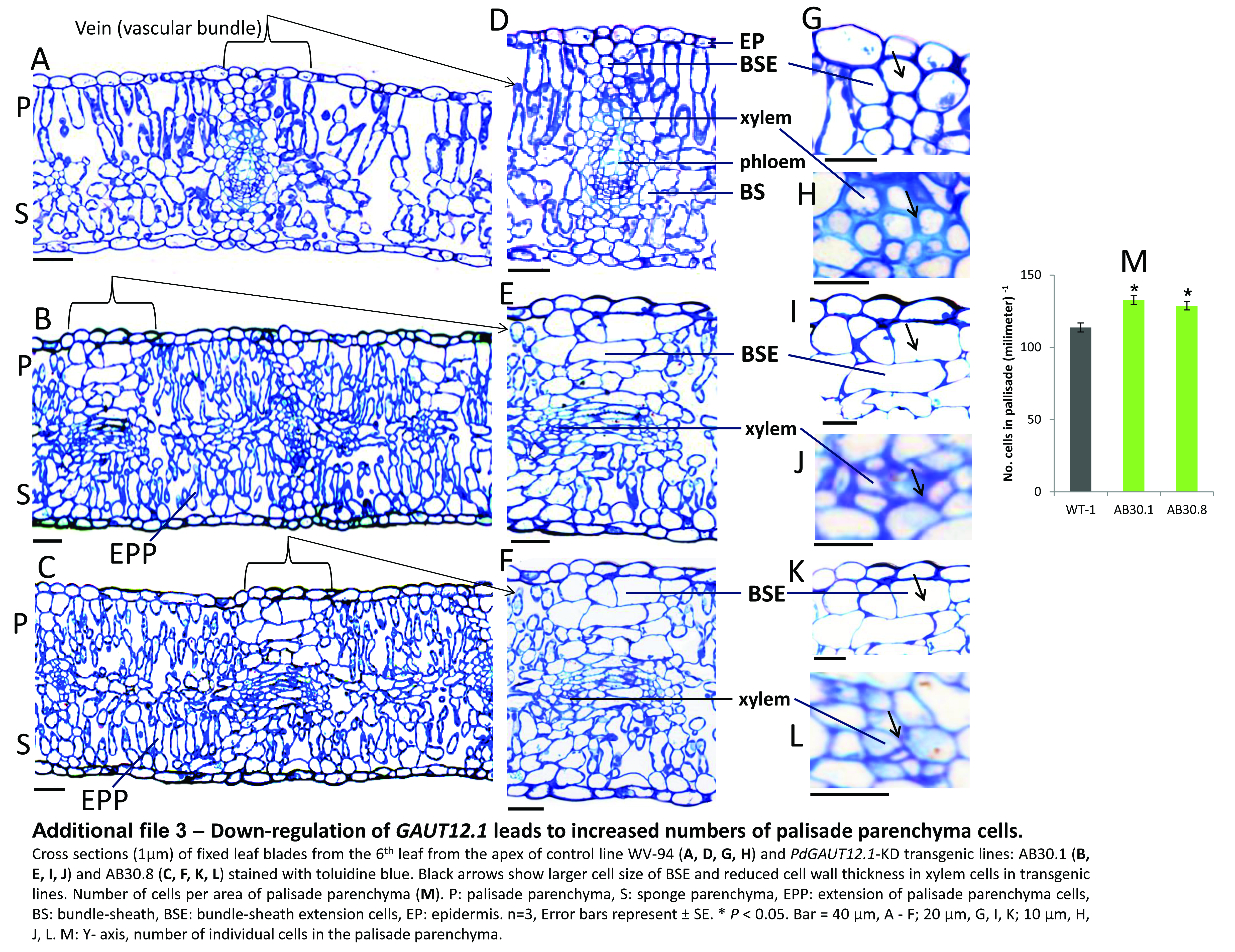

Supplement: Additional file 3: — Downregulation of GAUT12.1 leads to increased numbers of palisade parenchyma cells. [file 13068_2015_218_MOESM3_ESM.tiff]

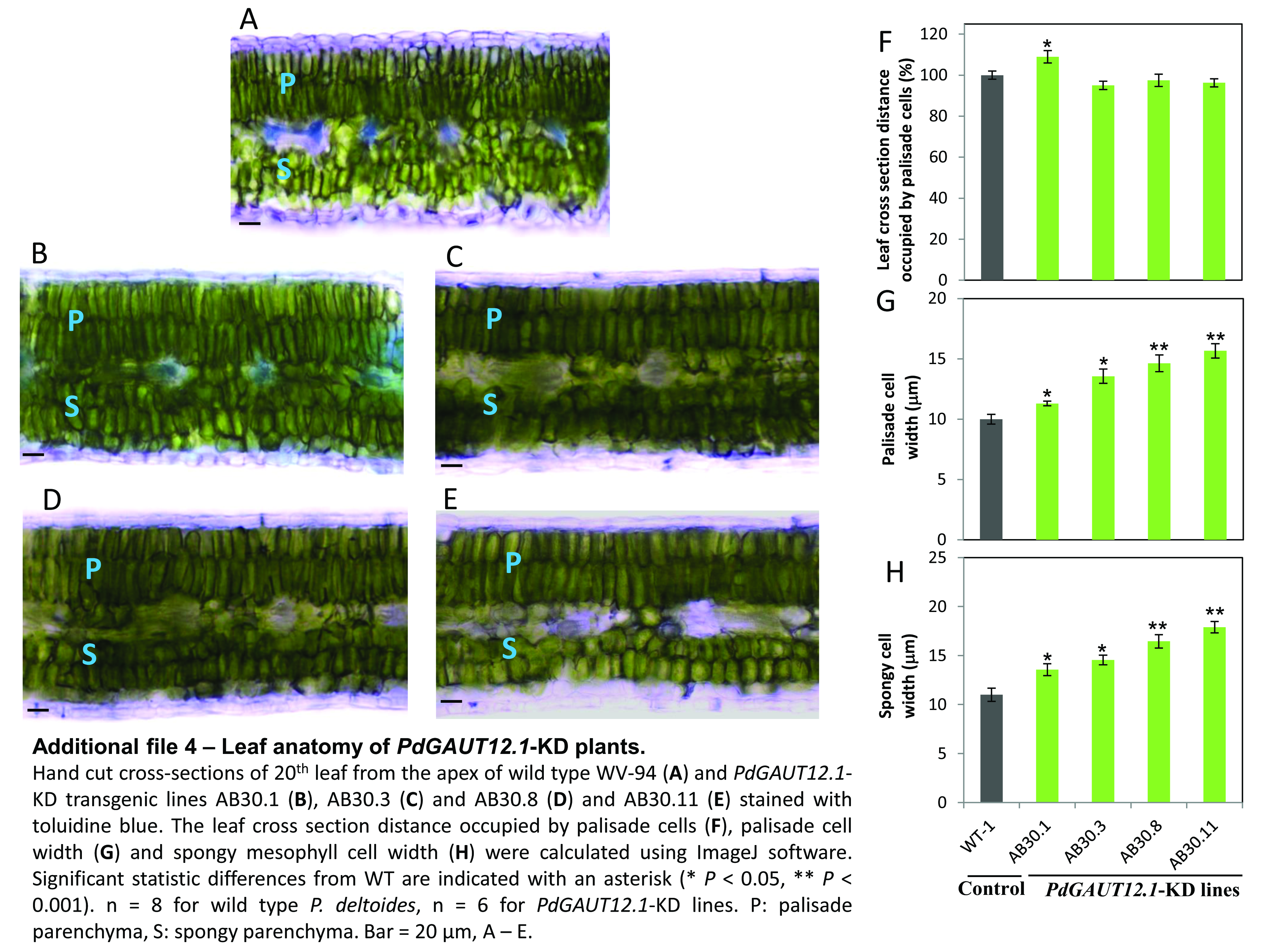

Supplement: Additional file 4: — Leaf anatomy of PdGAUT12.1 -KD plants. [file 13068_2015_218_MOESM4_ESM.tiff]

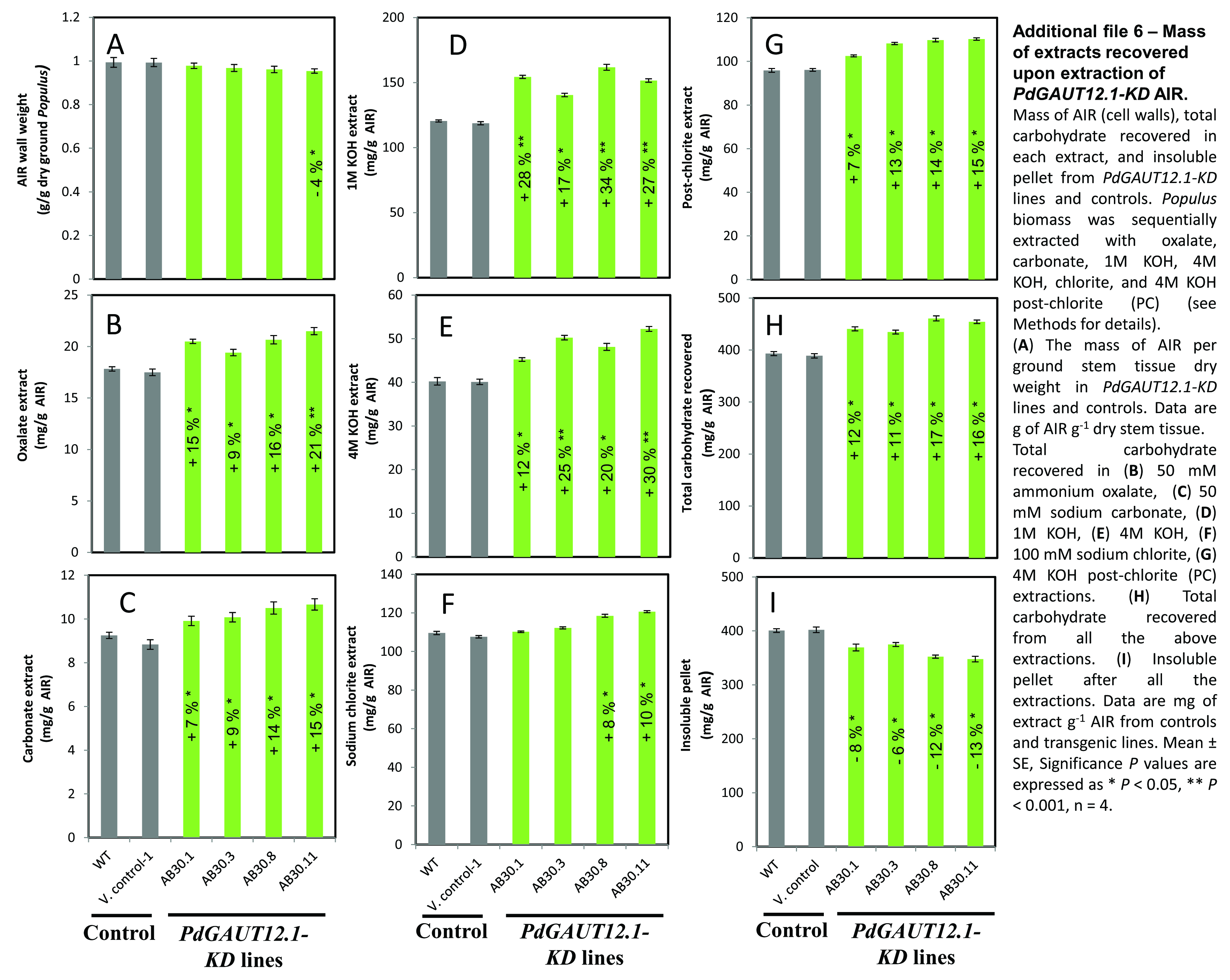

Supplement: Additional file 6: — Mass of extracts recovered upon extraction of PdGAUT12.1 -KD AIR. [file 13068_2015_218_MOESM6_ESM.tiff]
