# Supplementary material for: Downregulation of GAUT12 in Populus deltoides by RNA silencing results in reduced recalcitrance, increased growth and reduced xylan and pectin in a woody biofuel feedstock
Source: Biotechnol Biofuels. 2015 Mar 12;8:41. doi: 10.1186/s13068-015-0218-y (PMC4369864; doi:10.1186/s13068-015-0218-y)
Supplement: Additional file 5: — List of primers used for complementation study. [file 13068_2015_218_MOESM5_ESM.docx]

**Additional file 5 – List of primers used for complementation study.**

| Primer name | Primer sequences |
| --- | --- |
| GAUT12-Bam | aatcg**ggatcc**ATGCAGTTACATATATCTCCG |
| GAUT12-Apa | ccatt**gggccc**TCATGATGCTCTAATGTGAC |
| PdGAUT12.1-F | cgctgtgttgtgtgctcg |
| PdGAUT12.1-R | ttctggattcaaggccttgg |
| PdGAUT12.2-F | caagaaactgctctacttttggg |
| PdGAUT12.2-R | ccaccattgcggtcaacg |
| PdGAUT12-Bam | cg**ggatcc**ATGCAGCTTCATATATCACCAAG |
| PdGAUT12-Apa | tt**gggccc**TCATGTCCTAATATGACAGCCC |

Coding sequences in capital letters, BamHI and ApaI restriction sites bold; the same 2^nd^ round primers were used for *PdGAUT12*.1 and *PdGAUT12.2*, because the two genes had identical sequences surrounding the start and stop codon.
