# Supplementary material for: Downregulation of GAUT12 in Populus deltoides by RNA silencing results in reduced recalcitrance, increased growth and reduced xylan and pectin in a woody biofuel feedstock
Source: Biotechnol Biofuels. 2015 Mar 12;8:41. doi: 10.1186/s13068-015-0218-y (PMC4369864; doi:10.1186/s13068-015-0218-y)
Supplement: Additional file 7: — Monosaccharide composition and total carbohydrate of cell wall fractions from PdGAUT12.1 -KD and control plants. [file 13068_2015_218_MOESM7_ESM.docx]

**Additional file 7 – Monosaccharide composition and total carbohydrate of cell wall fractions from *PdGAUT12.1*-KD and control plants**.

Monosaccharide composition and total carbohydrate in oxalate, carbonate, 1M KOH, 4M KOH, Chlorite and Post Chlorite 4M KOH extracts of AIR from WT and *PdGAUT12.1*-KD lines. Values in bold are reduced in amount compared to WT in extract listed.

| **Plant**  **type** | **Mass of designated carbohydrate (µg) per mg of each designated extract** | | | | | | | | | | **Extract** |
| --- | --- | --- | --- | --- | --- | --- | --- | --- | --- | --- | --- |
|  | **Ara** | **Rha** | **Fuc** | **Xyl** | **GlcA** | **GalA** | **Man** | **Gal** | **Glc** | **Total** |  |
| WT | 4.05 | 0.95 | 0.14 | 2.26 | 0.05 | 5.05 | 3.08 | 2.64 | 5.64 | 23.86 | Oxalate |
| AB30.1 | **3.72** | **0.04** | 0.04 | 3.18 | 0.07 | **2.63** | 6.21 | 5.40 | 18.16 | 39.45 |  |
| AB30.3 | **3.59** | **0.22** | 0.05 | 3.12 | 0.07 | **2.64** | 6.00 | 5.37 | 16.11 | 37.17 |  |
| AB30.8 | **3.75** | **0.23** | 0.11 | 3.61 | 0.08 | **1.29** | 6.75 | 5.76 | 11.02 | 32.60 |  |
| AB30.11 | **3.65** | **0.26** | 0.07 | 3.71 | 0.09 | **1.98** | 6.48 | 6.09 | 12.52 | 34.85 |  |
| WT | 0.87 | 0.41 | 0.04 | 3.66 | 0.11 | 2.54 | 1.49 | 0.77 | 2.77 | 12.66 | Carbonate |
| AB30.1 | **0.28** | **0.18** | 0.06 | 4.34 | 0.15 | **1.99** | 2.14 | 0.91 | 4.75 | 14.80 |  |
| AB30.3 | **0.37** | **0.21** | 0.05 | 4.55 | 0.16 | **1.61** | 2.12 | 1.07 | 5.39 | 15.53 |  |
| AB30.8 | **0.41** | **0.27** | 0.08 | 4.91 | 0.19 | **0.46** | 2.48 | 1.09 | 6.01 | 15.90 |  |
| AB30.11 | **0.39** | **0.28** | 0.06 | 4.93 | 0.18 | **0.95** | 2.20 | 0.96 | 5.98 | 15.83 |  |
| WT | 3.11 | 8.63 | 0.94 | 544.18 | 47.68 | 11.33 | 10.52 | 5.41 | 16.97 | 648.77 | 1M KOH |
| AB30.1 | 4.41 | **6.51** | 1.26 | **480.01** | **39.60** | **5.72** | 93.09 | 55.73 | 172.05 | 858.38 |  |
| AB30.3 | 4.01 | **4.85** | 1.70 | **413.51** | **32.13** | **4.72** | 94.37 | 57.56 | 210.00 | 822.85 |  |
| AB30.8 | 5.27 | **2.86** | 1.39 | **413.76** | **31.71** | **3.66** | 100.74 | 72.77 | 211.00 | 843.16 |  |
| AB30.11 | 6.24 | **6.62** | 1.17 | **396.27** | **29.17** | **4.32** | 102.16 | 63.92 | 207.89 | 817.76 |  |
| WT | 1.70 | 2.21 | 0.36 | 146.01 | 18.11 | 2.08 | 6.18 | 2.43 | 13.28 | 192.36 | 4M KOH |
| AB30.1 | **1.15** | **1.76** | **0.29** | **123.90** | **15.42** | **1.35** | 24.20 | 15.31 | 44.89 | 228.27 |  |
| AB30.3 | **1.71** | **1.84** | **0.22** | **121.63** | **15.00** | **1.35** | 37.41 | 20.01 | 62.98 | 262.15 |  |
| AB30.8 | **1.25** | **1.57** | **0.21** | **106.13** | **13.39** | **0.73** | 40.55 | 16.81 | 76.18 | 256.82 |  |
| AB30.11 | **1.63** | **1.99** | **0.35** | **117.86** | **14.68** | **0.84** | 36.14 | 18.56 | 87.64 | 279.69 |  |
| WT | 13.93 | 5.87 | 0.00 | 30.3 | 0.00 | 9.15 | 2.51 | 31.61 | 146.43 | 239.80 | Chlorite |
| AB30.1 | **8.20** | **3.03** | 0.00 | **19.53** | 0.00 | **6.02** | 9.34 | 47.24 | 197.56 | 290.92 |  |
| AB30.3 | **7.74** | **3.32** | 0.00 | **19.31** | 0.00 | **5.73** | 8.43 | 45.85 | 230.53 | 320.91 |  |
| AB30.8 | **7.06** | **1.62** | 0.00 | **15.88** | 0.00 | **4.69** | 12.61 | 61.57 | 254.02 | 357.45 |  |
| AB30.11 | **7.51** | **3.60** | 0.47 | **18.05** | 0.00 | **5.61** | 11.86 | 54.93 | 254.27 | 356.30 |  |
| WT | 1.17 | 4.31 | 0.11 | 147.59 | 12.60 | 6.02 | 8.02 | 6.76 | 17.26 | 203.84 | 4M KOH PC |
| AB30.1 | **1.15** | **3.98** | **0.08** | **113.75** | **9.97** | **5.65** | 32.71 | 29.92 | 41.28 | 238.49 |  |
| AB30.3 | **1.12** | **4.07** | **0.06** | **109.53** | **9.39** | **5.53** | 41.12 | 36.64 | 65.38 | 272.84 |  |
| AB30.8 | **1.05** | **3.69** | **0.06** | **88.49** | **8.07** | **5.33** | 45.71 | 39.47 | 75.03 | 266.90 |  |
| AB30.11 | **1.15** | **4.07** | **0.09** | **98.31** | **8.71** | **5.13** | 44.56 | 37.93 | 83.60 | 283.55 |  |
