# Supplementary material for: Downregulation of GAUT12 in Populus deltoides by RNA silencing results in reduced recalcitrance, increased growth and reduced xylan and pectin in a woody biofuel feedstock
Source: Biotechnol Biofuels. 2015 Mar 12;8:41. doi: 10.1186/s13068-015-0218-y (PMC4369864; doi:10.1186/s13068-015-0218-y)
Supplement: Additional file 9: — Lignin peak and precursor assignments of analytical pyrolysis mass spectra. [file 13068_2015_218_MOESM9_ESM.docx]

**Additional file 9 – Lignin peak and precursor assignments of analytical pyrolysis mass spectra.**

Peak and precursor assignments in mass spectra of lignified samples**^a^**.


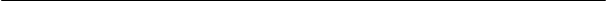


m/z Assignment Precursor


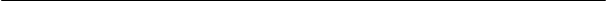


94 phenol H, S, G

120 vinylphenol H

124 guaiacol G

137 ethylguaiacol, homovanillin, coniferyl alcohol G

138 methylguaiacol G

150 vinylguaiacol G

154 syringol S

164 allyl propenyl guaiacol G

167 ethylsyringol, syringylacetone, propiosyringone S

168 4-methyl-2,6-dimethoxyphenol S

178 coniferyl aldehyde G

180 coniferyl alcohol, vinylsyringol S, G

182 syringaldehyde S

194 4-propenylsyringol S

208 sinapylaldehyde S

210 sinapylalcohol S


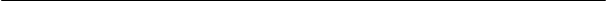


^a^Evans RJ, Milne TA: Molecular characterization of the pyrolysis of biomass. *Energy Fuels* 1987, 1:123-137.
